# Home Time Following Emergency Department Visits Among People With Dementia

**DOI:** 10.1001/jamanetworkopen.2025.49154

**Published:** 2025-12-29

**Authors:** Justine Seidenfeld, Lindsay Zepel, Valerie A. Smith, Fernanda Bellolio, Anita A. Vashi, Courtney H. Van Houtven, Susan N. Hastings

**Affiliations:** 1 Center of Innovation to Accelerate Discovery and Practice Transformation (ADAPT), Durham Veterans Affairs Health Care System, Durham, North Carolina; 2 Department of Emergency Medicine, Duke University School of Medicine, Durham, North Carolina; 3 Department of Population Health Sciences, School of Medicine, Duke University, Durham, North Carolina; 4 Department of Biostatistics and Bioinformatics, School of Medicine, Duke University, Durham, North Carolina; 5 Division of General Internal Medicine, Department of Medicine, School of Medicine, Duke University, Durham, North Carolina; 6 Department of Emergency Medicine, Mayo Clinic, Rochester, Minnesota; 7 Department of Medicine (Geriatrics), Mayo Clinic, Rochester, Minnesota; 8 Division of Health Care Policy and Research, Mayo Clinic, Rochester, Minnesota; 9 Center for Innovation to Implementation, Palo Alto VA Medical Center, Palo Alto, California; 10 Department of Emergency Medicine, Palo Alto VA Medical Center, Palo Alto, California; 11 Department of Emergency Medicine, Stanford University, Stanford, California; 12 Duke-Margolis Institute for Health Policy, Duke University, Durham, North Carolina

## Abstract

**Importance:**

Emergency department (ED) visits for people with dementia often involve care (including the admission or discharge decision) with lasting effects on quality of life. Home time—a claims-based measure of days alive and outside of institutional care—provides a patient-centered way to assess downstream outcomes of ED care in this population.

**Objective:**

To examine variations in home time following an ED visit by patient, facility, and visit characteristics among veterans with dementia.

**Design, Setting, and Participants:**

This national retrospective cohort study used US Department of Veterans Affairs (VA) health care system data sources. Participants were veterans aged 65 to 110 years with dementia with an eligible Veterans Health Administration ED visit in fiscal years 2017 (October 1, 2016, through September 30, 2017) and/or 2018 (October 1, 2017, through September 30, 2018). Data were analyzed from May 1, 2024, to May 15, 2025.

**Exposures:**

Patient, ED facility, and ED visit factors.

**Main Outcomes and Measures:**

Home time, operationalized for modeling as days not at home in the 180 days after the index ED visit. Associations between patient, ED facility, and ED visit characteristics and home time were assessed using a negative binomial model.

**Results:**

The study included 51 707 veterans with dementia (mean [SD] age, 79.9 [8.59] years; 97.6% male; 52.2% married). The mean (SD) number of days not at home within 180 days was 21.7 (34.5). Of the total cohort, 4.5% never returned home in the 180 days after their ED visit, while 18.2% had all subsequent days at home. ED admission (vs discharge) disposition was the factor associated with the largest increase in days not at home (rate ratio [RR], 3.18; 95% CI, 3.08-3.29). Patient factors associated with more days not at home included very high frailty (RR, 1.27; 95% CI, 1.21-1.33), unhoused status (RR, 1.50; 95% CI, 1.39-1.63), unmarried status (never married: RR, 1.24 [95% CI, 1.16-1.32]; divorced, separated, or widowed: RR, 1.24 [95% CI, 1.20-1.28]), and depression (RR, 1.13; 95% CI, 1.09-1.17).

**Conclusions and Relevance:**

The findings of this cohort study suggest that home time offers a patient-centered measure of post-ED outcomes for individuals with dementia. Future work should refine its application, particularly regarding the ED disposition decision, to better evaluate the long-term impact of ED care decisions.

## Key Points

**Question:**

Among veterans with dementia, what are the patterns of home time—days outside of care settings—after an emergency department (ED) visit, and what factors are associated with variation?

**Findings:**

In this cohort study including 51 707 veterans, ED admission (vs discharge) disposition was associated with the largest decrease in home time, and admitted patients experienced substantial health care use following the initial care episode. Frailty, being unhoused, being unmarried, and depression were associated with decreased home time.

**Meaning:**

The findings suggest home time offers a patient-centered measure of post-ED quality of life, but refinements are needed to account for the initial disposition.

## Teaser

This cohort study examines patterns of home time, assessed as days spent outside of institutional care settings, following an emergency department visit among veterans aged 65 years or older with dementia.

## Introduction

People with dementia experience significantly higher rates of acute care use than those without dementia, including twice as many emergency department (ED) visits, ED revisits, and subsequent inpatient stays.^1,2,3,4^ ED disposition decisions—that is, determining whether to admit someone to the hospital or discharge them home—for people with dementia are often complex and uncertain.^5,6^ While inappropriate discharges can lead to worsening morbidity and recurrent ED use, hospital admissions can lead to functional decline and prolonged long-term care.^3,7,8^ Recognizing this, EDs are increasingly positioned as part of the continuum of age-friendly care, with efforts to enhance care transitions and incorporate screening and interventions for geriatric syndromes such as falls and delirium.^9,10^ ED visits thus represent sentinel events for people with dementia, with lasting implications for quality of life and well-being.

However, evaluating the downstream impact of ED care remains challenging. The Geriatric Emergency Care Applied Research Network 2.0–Advancing Dementia Care (GEAR 2.0) program highlighted the need for better patient-centered outcome measures to assess the effects of ED interventions for people with dementia.^11,12^ Home time, defined as days alive and outside of acute or postacute care settings, has emerged as a promising quality-of-life metric in other settings.^13,14^ Home time is especially relevant for people with dementia, who overwhelmingly express a preference to remain at home whenever possible and for whom traditional outcome measures may be difficult to capture.^15,16,17,18^

Despite growing interest, the application of home time to ED care is novel and methodologically complex, with questions including what follow-up windows are optimal and how to account for postacute institutional care.^19^ One known study examining home time after an ED visit found that Medicare beneficiaries with dementia had fewer days at home within 30 days postvisit compared with those without dementia.^20^ However, a 30-day window may underestimate longer-term impacts, such as postacute skilled nursing facility (SNF) stays and long-term care.

To address this gap, we conducted a national cohort study of veterans with dementia who had an ED visit within the Veterans Health Administration (VHA). Our objectives were to describe patterns of home time in the 180 days following an ED visit and assess variations by patient, facility, and ED visit factors. The VHA’s comprehensive claims data offer a unique opportunity to examine ED visits and their outcomes in a large population of people with dementia.

## Methods

### Study Design and Setting

This retrospective cohort study was approved by the Durham Veterans Affairs institutional review board, which waived informed consent as this was secondary research with no interactions of interventions with participants. We followed the Strengthening the Reporting of Observational Studies in Epidemiology (STROBE) reporting guideline.^21^

### Data Sources

Department of Veterans Affairs (VA) and Centers for Medicare & Medicaid Services (CMS) administrative data sources, including the VA Corporate Data Warehouse (CDW) data repository,^22^ were used to define veteran sociodemographics, clinical characteristics, and outcomes. The primary outcome measure of home time was derived from the VA Residential History File (RHF),^23^ which is constructed using data from several VA and CMS sources (VA, Medicare Fee-for-Service, Medicare Advantage, and Medicaid) to identify daily health service use and location of care.^22^ *International Classification of Diseases, Ninth Revision (ICD-9)* or *International Statistical Classification of Diseases and Related Health Problems, 10th Revision (ICD-10)* diagnosis codes for a dementia diagnosis were identified in both VA data (inpatient and outpatient files) and Medicare data (outpatient, carrier, home health, SNF, hospice, and Medicare Provider Analysis and Review summary files). All other patient baseline variables were drawn from the VA CDW.

### Study Population

Our study sample included VHA users who had at least 1 qualifying VHA ED visit in fiscal year (FY) 2017 (October 1, 2016, through September 30, 2017) and/or FY 2018 (October 1, 2017, through September 30, 2018) and were 65 to 110 years of age at the time of their ED visit. Of these, veterans with dementia were defined as those with 2 or more *ICD-9* or *ICD-10* diagnoses of dementia recorded in the 2 years before their ED visit (eTable 1 in Supplement 1).^24^

### Index ED Visits

Potentially eligible index ED visits were defined using the CDW, including ED visits in which the patient was admitted to the hospital or discharged home. To ensure home time reflected variation in post-ED care needs and recovery trajectories, we excluded visits for conditions with a clearly emergent clinical course or expected admission. Specifically, we excluded ED visits that were (1) triaged as Emergency Severity Index level 1 (highest acuity); (2) admitted directly to intensive care unit (ICU)–level care or hospice; or (3) associated with urgent procedures within 48 hours (eg, appendectomy or coronary artery bypass graft), based on prior work characterizing acute hospital admissions.^25^ We also excluded visits where the patient died on the day of or day after the ED visit, left without being seen or against medical advice, or was transferred to another facility, as these transfers are typically for specialized services, likely requiring urgent admission. In addition, we excluded ED visits for veterans already in institutional settings (eg, nursing homes) prior to the ED visit, as discharge to home was not applicable. For veterans with multiple eligible index ED visits, we selected the first in our study period (October 1, 2016, to September 30, 2018). Visits without corresponding records in the RHF, which was used to calculate outcomes, were excluded.

### Baseline Variables and Model Covariates

Patient-level baseline variables were selected based on their potential to modify home time, including demographics such as age, sex, race, ethnicity, geocoded location, marital status, housing status, and depression. We used clinical and health care use variables such as frailty and service connection (described in eTable 2 in Supplement 1), and to assess the robustness of the results, we did additional sensitivity analyses, adding VA priority group to capture financial status and adding a variable for any hospitalizations in the prior year. Race and ethnicity data are collected through self-report when veterans apply for health benefits and could also be collected at the time of any inpatient or outpatient visit to a VA facility. Race categories were American Indian or Alaska Native, Asian, Black or African American, Native Hawaiian or Other Pacific Islander, White, and multiracial, and ethnicity categories were Hispanic and non-Hispanic. We also included facility-level variables such as facility rurality, facility complexity, and annual ED volume (eTable 2 in Supplement 1). To identify clinically meaningful subgroups of ED visits, we categorized index ED visits into 5 groups based on discharge diagnosis *ICD-10* codes. As a sensitivity analysis, we also tested an alternative classification using patient-reported chief concerns instead of clinician-assigned discharge diagnoses (eTable 3 in Supplement 1). All patient-level variables were based on a 1-year lookback from the index ED visit date, and all ED facility–level variables were based on the fiscal year of the ED visit.

### Primary Outcome

The primary outcome was home time, operationalized for modeling as days not at home in the 180 days after the index ED visit; this timeline was selected based on prior work with a home time outcome measure in the VHA.^26^ We also considered home time using 90 days and 365 days in sensitivity analyses, using the same analytic approach. For each, the count of days started the day after the ED visit occurred. Home time was defined as days alive and not in an ED, inpatient setting, postacute or SNF setting, or nursing home setting (including a VA Community Living Center, State Veterans Home, or Medicaid long-term stay). Days at home included those with outpatient visits, home health or respite, and hospice in all settings. We considered inpatient hospice to be consistent with a higher-quality-of-life day,^14,27^ and for completeness, we reported the rate of patients with any inpatient hospice stay. If patients had more than 100 cumulative days of a non–hospice-associated nursing home or SNF episode (which could include intervening inpatient or ED visits but not interruptions by a stay in a home setting), we deemed it a partial follow-up period and censored follow-up at 100 days.^28^ This time frame was selected because it aligned with Medicare payment policies for postacute care and because patients in a nursing facility for more than 100 days are most likely to remain there long term, and thus home time as a concept is inherently different. For patients who (1) died or (2) had a partial follow-up period ending with 100 days in a nursing home or SNF setting, their maximum number of days (180 for the primary outcome and 90 and 365 for each of the sensitivity analyses) for the home time calculation was the number of days they were alive or not censored, respectively. There were no other reasons for partial follow-up and no missing data (eTable 2 in Supplement 1).

### Secondary Outcomes

Secondary outcomes included any ED revisit within 30 days of the index ED visit (VA or community ED) and 30-day mortality. We censored data for patients at the earliest of (1) 30 days after the index ED visit, (2) the end of data availability (December 31, 2019), or (3) the outcome of an ED revisit or death.

### Statistical Analysis

For descriptive data, we calculated frequencies with percentages for categorical variables and means with SDs or medians with IQRs for continuous variables. For the primary outcome of home time at 180 days, the association between explanatory variables of interest and home time was assessed using a negative binomial model with the outcome of days not at home and including an offset of log(days observed) to account for different follow-up times. Days not at home were calculated by subtracting the days at home from the maximum number of days in the period. VA Medical Center (VAMC)–level random effects were included to account for correlation among patients at the same VAMC. To analyze chief concerns, we used effect coding to compare the mean days of nonhome time within each chief concern category for the average veteran. The primary analysis was inclusive of all ED visits, and we additionally elected to stratify by disposition. For the secondary outcome of 30-day ED revisits, we calculated incidence based on the cumulative-incidence function to account for the competing risk of mortality. The Kaplan-Meier method was used to calculate the rate of all-cause mortality. Cox proportional hazards regression was used to assess the association between secondary outcomes and the variables of interest, censoring for the competing event of death in the ED revisit model to estimate the cause-specific hazard.^29,30^ A robust sandwich estimator was used to account for correlation among patients at the same VAMC. The analysis was performed from May 1, 2024, to May 15, 2025, using SAS Enterprise Guide, version 8.3 (SAS Institute), and SQL Server Management Studio, version 20 (Microsoft).

## Results

### Study Population

**Figure 1.**
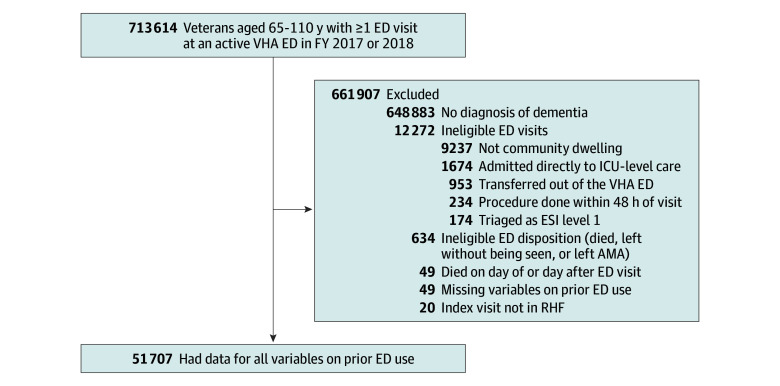
Study Cohort Flow Diagram AMA indicates against medical advice; ED, emergency department; ESI, Emergency Severity Index; FY, fiscal year; ICU, intensive care unit; RHF, Residential History File; VHA, Veterans Health Administration.

We identified 713 614 veterans between 65 and 110 years of age with at least 1 visit at a VHA ED between FY 2017 and FY 2018. Of these, 64 731 (9.1%) had a dementia diagnosis. After applying exclusions based on visit acuity, disposition, residential setting, and data completeness, the final analytic cohort included 51 707 veterans with an eligible VHA ED visit (Figure 1). This cohort was predominantly male (97.6%; 2.4% female), and mean (SD) age was 79.9 (8.59) years. A total of 0.6% of the veterans were American Indian or Alaska Native, 0.5% were Asian, 21.1% were Black or African American, 0.7% were Native Hawaiian or Other Pacific Islander, 73.0% were White, 0.8% were multiracial, and 3.3% had unknown or missing race; 9.5% were Hispanic, 88.4% were non-Hispanic, and 2.1% had unknown ethnicity. Most were urban dwelling (74.4%), and a small majority were married (52.2%). There were 20 443 (39.5%) admitted on their index ED visit. The Table details baseline sociodemographic, ED facility, and ED visit characteristics used in the home time models.

**Table.** Descriptive Characteristics of Patients, ED Visits, and ED Facilities, Total and by Disposition Decision

Characteristic^b^	Patients^a^			SMD, %^c^
	Total (N = 51 707)	Discharged (n = 31 264)	Admitted (n = 20 443)	
**Patient**				
Age, mean (SD), y	79.9 (8.59)	79.5 (8.52)	80.5 (8.66)	11.1
Sex				
Female	1230 (2.4)	784 (2.5)	446 (2.2)	2.2
Male	50 477 (97.6)	30 480 (97.5)	19 997 (97.8)	
Ethnicity				
Hispanic	4916 (9.5)	3105 (9.9)	1811 (8.9)	6.0
Non-Hispanic	45 728 (88.4)	27 603 (88.3)	18 125 (88.7)	
Unknown	1063 (2.1)	556 (1.8)	507 (2.5)	
Race				
American Indian or Alaska Native	319 (0.6)	197 (0.6)	122 (0.6)	6.0
Asian	249 (0.5)	155 (0.5)	94 (0.5)	
Black or African American	10 885 (21.1)	6536 (20.9)	4349 (21.3)	
Native Hawaiian or Other Pacific Islander	369 (0.7)	218 (0.7)	151 (0.7)	
White	37 760 (73.0)	23 005 (73.6)	14 755 (72.2)	
Multiracial	394 (0.8)	235 (0.8)	159 (0.8)	
Unknown or missing race	1731 (3.3)	918 (2.9)	813 (4.0)	
Rurality				
Urban	38 488 (74.4)	23 147 (74.0)	15 341 (75.0)	4.0
Rural	12 161 (23.5)	7579 (24.2)	4582 (22.4)	
Insular or highly rural	407 (0.8)	235 (0.8)	172 (0.8)	
Missing	651 (1.3)	303 (1.0)	348 (1.7)	
Marital status				
Married	26 981 (52.2)	16 901 (54.1)	10 080 (49.3)	10.0
Never married	3284 (6.4)	1902 (6.1)	1382 (6.8)	
Divorced, separated, or widowed	21 054 (40.7)	12 231 (39.1)	8823 (43.2)	
Unknown	388 (0.8)	230 (0.7)	158 (0.8)	
Driving distance to nearest VAMC, km				
Median (IQR)	14.5 (6.4-27.4)	14.5 (8.0-29.0)	14.5 (6.4-27.4)	3.7
≤64	47 807 (92.5)	28 998 (92.8)	18 809 (92.0)	5.6
>64	2163 (4.2)	1339 (4.3)	824 (4.0)	
Missing	1737 (3.4)	927 (3.0)	810 (4.0)	
ADI				
Median (IQR)	57 (35.0-79.0)	57 (35.0-79.0)	56 (34.0-79.0)	2.1
Quartile				
0-25 (Lowest deprivation)	8000 (15.5)	4706 (15.1)	3294 (16.1)	5.9
26-50	13 521 (26.1)	8257 (26.4)	5264 (25.7)	
51-75	14 285 (27.6)	8720 (27.9)	5565 (27.2)	
76-100	14 356 (27.8)	8752 (28.0)	5604 (27.4)	
Missing	1545 (3.0)	829 (2.7)	716 (3.5)	
JEN Frailty Index^d^				
Median (IQR)	6 (4.0-8.0)	6 (4.0-8.0)	6 (4.0-8.0)	4.4
0-3	8499 (16.4)	5044 (16.1)	3455 (16.9)	13.3
4-5	13 283 (25.7)	8447 (27.0)	4836 (23.7)	
6-7	14 595 (28.2)	9093 (29.1)	5502 (26.9)	
≥8 (Very high frailty)	13 478 (26.1)	7770 (24.9)	5708 (27.9)	
Missing	1852 (3.6)	910 (2.9)	942 (4.6)	
Unhoused	2083 (4.0)	1264 (4.0)	819 (4.0)	0.2
Service connected, %^e^				
0-49	8775 (17.0)	5405 (17.3)	3370 (16.5)	10.2
50-99	8938 (17.3)	5753 (18.4)	3185 (15.6)	
100	7062 (13.7)	4409 (14.1)	2653 (13.0)	
No	26 932 (52.1)	15 697 (50.2)	11 235 (55.0)	
Depression				
Known or likely	20 667 (40.0)	12 032 (38.5)	8635 (42.2)	10.5
None	24 107 (46.6)	15 220 (48.7)	8887 (43.5)	
Missing	6933 (13.4)	4012 (12.8)	2921 (14.3)	
**Facility**				
Rurality				
Urban	49 732 (96.2)	29 869 (95.5)	19 863 (97.2)	8.7
Rural	1975 (3.8)	1395 (4.5)	580 (2.8)	
Facility complexity score				
1a (Most complex)	26 590 (51.4)	15 049 (48.1)	11 541 (56.5)	16.7
1b, 1c, 2, or 3	25 117 (48.6)	16 215 (51.9)	8902 (43.5)	
Annual ED volume, No.				
Median (IQR)	23 347 (16 096-28 417)	23 239 (15 927-28 417)	23 671 (16 784-28 027)	4.9
≤10 000	2342 (4.5)	1617 (5.2)	725 (3.5)	11.1
10 000 to ≤20 000	16 587 (32.1)	10 241 (32.8)	6346 (31.0)	
>20 000 to ≤30 000	22 336 (43.2)	12 951 (41.4)	9385 (45.9)	
>30 000	10 442 (20.2)	6455 (20.6)	3987 (19.5)	
**ED visit**				
*ICD-10* diagnosis category at index ED visit				
Chronic condition	10 531 (20.4)	5561 (17.8)	4970 (24.3)	49.4
Injury or MSK	7472 (14.5)	6280 (20.1)	1192 (5.8)	
Infection	6144 (11.9)	3486 (11.2)	2658 (13.0)	
Non-MSK	14 155 (27.4)	7231 (23.1)	6924 (33.9)	
Other	13 405 (25.9)	8706 (27.8)	4699 (23.0)	
Chief concern^f^				
Nonspecific concerns and geriatric syndromes	9608 (18.6)	4890 (15.6)	4718 (23.1)	38.1
Non-MSK symptoms	14 227 (27.5)	8191 (26.2)	6036 (29.5)	
Infection	3776 (7.3)	2472 (7.9)	1304 (6.4)	
MSK plus trauma	6227 (12.0)	4787 (15.3)	1440 (7.0)	
Psychiatric	1105 (2.1)	369 (1.2)	736 (3.6)	
Other	1409 (2.7)	1116 (3.6)	293 (1.4)	
<100 Visits in chief concern category	15 355 (29.7)	9439 (30.2)	5916 (28.9)	

Abbreviations: ADI, Area Deprivation Index; ED, emergency department; *ICD-10*, *International Statistical Classification of Diseases and Related Health Problems, Tenth Revision*; MSK, musculoskeletal; SMD, standardized mean differences comparing those admitted and discharged at their index ED visit; VAMC, Veterans Affairs Medical Center.

^a^ Data are presented as number (percentage) of patients unless otherwise indicated.

^b^ eTable 2 in Supplement 1 provides detailed descriptions of variables and specifications.

^c^ SMD greater than 10% represents a meaningful difference between the groups.

^d^ Score range 0 to 13, with higher scores indicating greater risk of institutional and home-based service use.

^e^ Measure is based on illness or injury related to military service and impacts health care benefits, co-payment rates, and disability compensation, with 0% being the least disabling and 100% the most disabling.

^f^ Chief concern categories were used in the sensitivity analysis model only.

### Days Not at Home After the Index ED Visit

**Figure 2.**
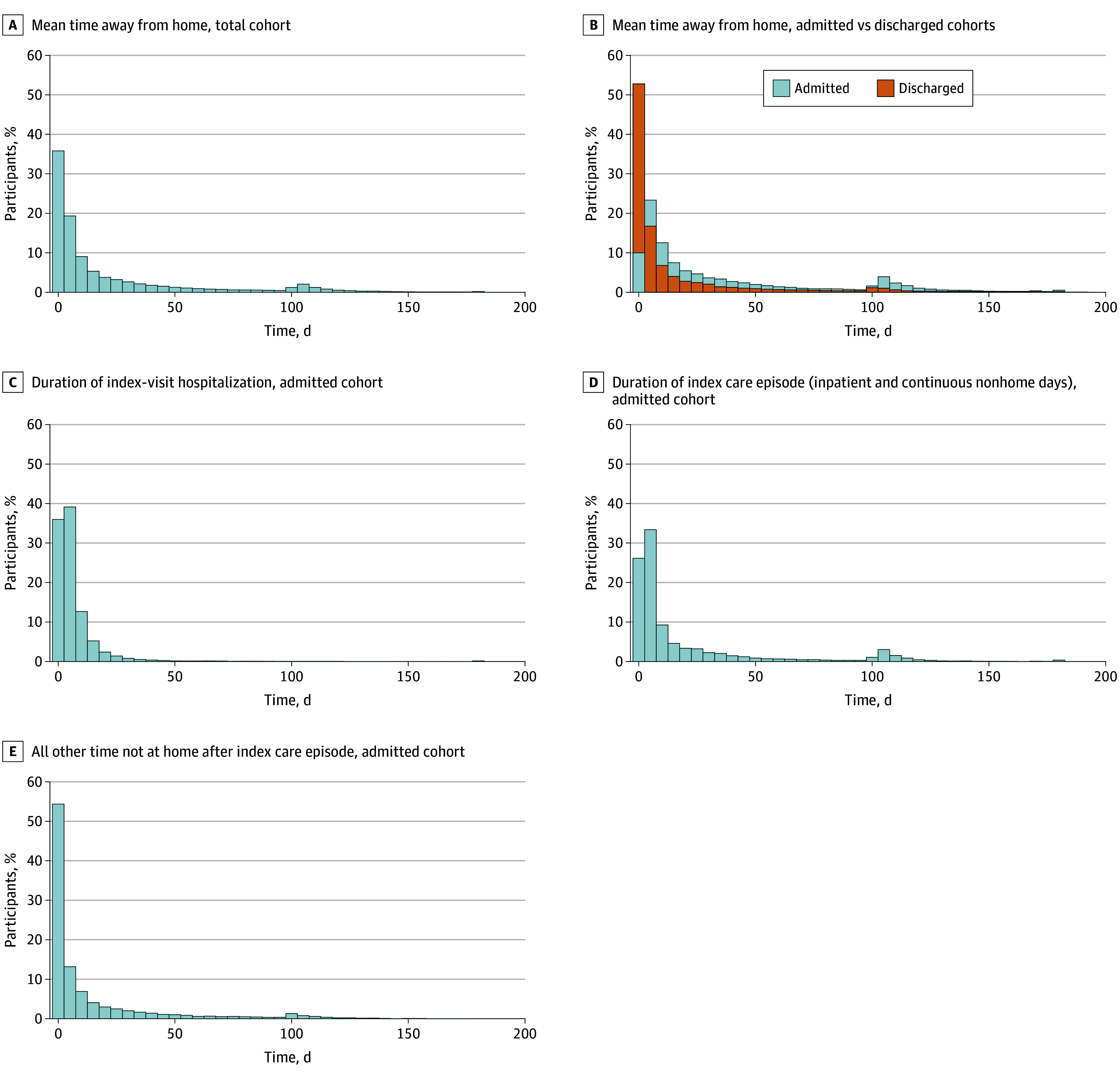
Descriptive Counts and Summary of Days Away From Home Within 180 Days After Emergency Department Visit

Across the cohort (Figure 2A), the mean (SD) number of days not at home in the 180 days after an ED visit was 21.7 (34.5). A total of 4.5% of individuals never returned home in the 180 days after their ED visit, while 18.2% had all subsequent days at home. eTable 4 in Supplement 1 provides additional description of distributions. A total of 7925 veterans (15.3%) died during the 180 days of follow-up, and 2777 (5.4%) were censored due to reaching 100 consecutive days in a nonhome setting; among those, 310 (11.2%) subsequently accrued additional days at home within the 180-day window that were not included in the analysis, representing 0.6% of individuals in the full analytic cohort. Although inpatient hospice days were counted as days at home to reflect higher quality of life, 736 veterans (1.4%) had at least 1 such day.

#### Discharged Patterns

Among veterans discharged home from the index ED visit (n = 31 264 [60.5%]), the mean (SD) number of subsequent days not at home was 13.6 (27.1) (Figure 2B). Notably, 9397 of those veterans (30.1%) had the maximum number of days at home after the ED visit. Additionally, 3007 (9.6%) died within the 180-day follow-up period, and 841 (2.7%) had more than 100 consecutive days not at home.

#### Admission Patterns

Among admitted veterans (n = 20 443 [39.5%]), the mean (SD) number of days not at home was 34.2 (40.5) (Figure 2B). The associated inpatient hospitalizations comprised a mean (SD) of 7.03 (12.5) days (Figure 2C), and the full episode of care (inclusive of the inpatient hospitalization and subsequent consecutive days not at home, such as at an SNF) comprised a mean (SD) of 20.1 (32.9) days (Figure 2D). The mean (SD) number of days not at home after the index episode of care was 14.1 (27.2) (Figure 2E). Among admitted patients, 2346 (11.5%) subsequently never returned to a home setting, either remaining hospitalized, dying before returning home, or being censored. Overall, 4918 veterans admitted during the index ED visit (24.1%) died in the 180-day follow up. Additionally, 1936 (9.5%) spent more than 100 consecutive days not at home at some point in the 180-day follow up period, which may have been preceded by days at home after the index ED visit.

### Characteristics Associated With Variation in Days Not at Home

**Figure 3.**
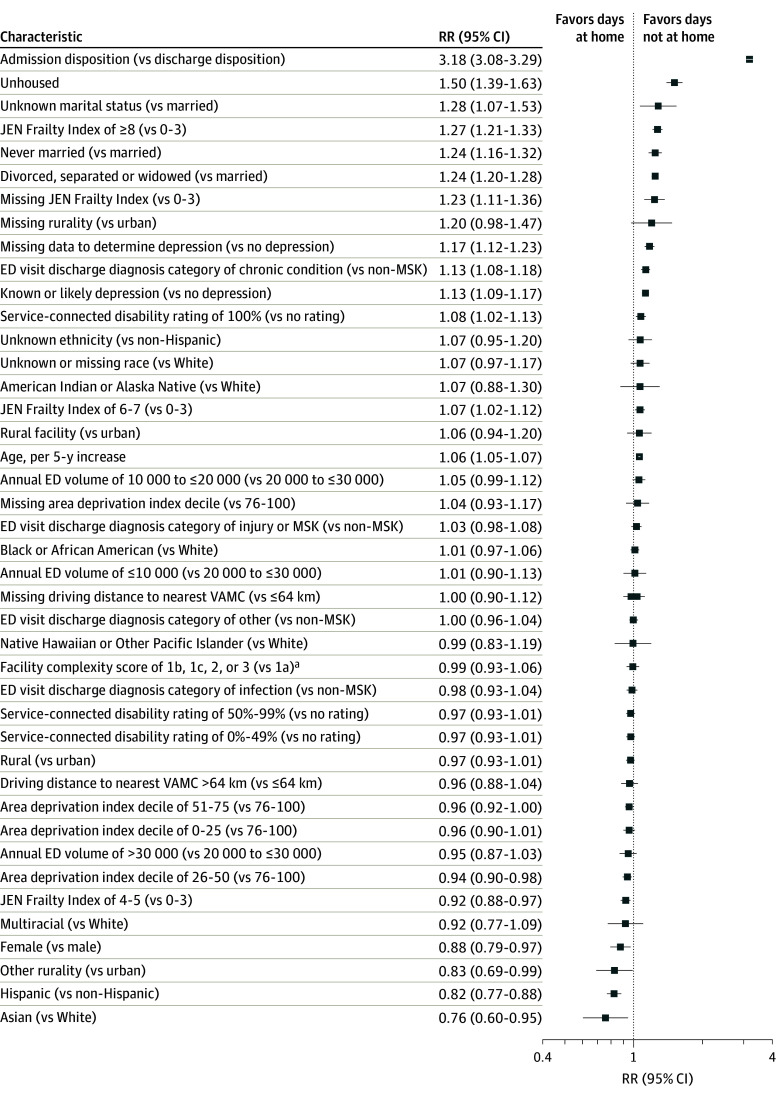
Association Between Baseline Characteristics and Days Away From Home (180 days), Total Cohort The JEN Frailty Index is a claims-based measure of frailty (score range, 0-13, with higher scores indicating greater risk of institutional and home-based service use). ED indicates emergency department; MSK, musculoskeletal; RR, rate ratio; VAMC, Veterans Affairs Medical Center. ^a^1a indicates most complex.

In adjusted models, ED admission disposition was the factor associated with the largest increase in days away from home (rate ratio [RR], 3.18; 95% CI, 3.08-3.29) (Figure 3). Other patient-level factors associated with more days away from home included unhoused status (RR, 1.50; 95% CI, 1.39-1.63), unmarried status (never married: RR, 1.24 [95% CI, 1.16-1.32]; divorced, separated, or widowed: RR, 1.24 [95% CI, 1.20-1.28]), very high frailty (RR, 1.27; 95% CI, 1.21-1.33), and depression (RR, 1.13; 95% CI, 1.09-1.17), along with older age and high service-connected disability rating. Asian race, Hispanic ethnicity, and female sex were associated with fewer days not at home (increased home time). In sensitivity analyses incorporating priority group and an indicator for prior hospitalizations, findings were consistent with our primary model. Specifically, the direction and statistical significance of other associations remained unchanged, suggesting model robustness. In these expanded models, veterans in the lower-priority group (ie, those required to pay co-payments) had fewer days away from home, while prior hospitalization was associated with more days away from home (eTable 5 in Supplement 1). No ED facility–level characteristics were significantly associated with days not at home. For ED visit–level characteristics, in addition to an admission disposition, an ED visit with a discharge diagnosis category related to a chronic condition was associated with increased days not at home. When stratified by admission status (eFigures 1 and 2 in Supplement 1), findings were largely consistent, although among admitted patients, injury- or musculoskeletal-related discharge diagnoses were also associated with increased days away from home (RR, 1.32; 95% CI, 1.23-1.42).

#### Characteristics by Outcome Duration and by Chief Concerns Associated With Variation in Days Not at Home

In the sensitivity analysis using different home time outcome durations (90 days, 180 days, and 365 days), there were few differences in the associations between patient, ED facility, or ED visit characteristics and subsequent health care use in the total cohort (eTable 6 in Supplement 1). In the sensitivity analysis using chief concerns, veterans with psychiatric concerns (eg, mental health, suicidal ideation, anxiety, depression, alcohol use, or hallucinations) had the largest RR of days not at home compared with the overall mean (RR, 1.31; 95% CI, 1.19-1.43; adjusted mean, 34.6 days [95% CI, 31.1-38.6 days]), followed by those with nonspecific concerns and geriatric syndromes (eg, falls, abnormal mental state, dizziness, confusion, dementia, or weakness) (RR, 1.19; 95% CI, 1.14-1.24; adjusted mean, 31.5 days [95% CI, 30.2-32.8 days]) (eTable 7 in Supplement 1).

### Secondary Outcomes

In the total cohort, 14 274 patients (27.6%) had a return visit to any VA or community ED within 30 days of the index visit, and 2065 (4.0%) died within 30 days of the index visit. eTable 8 in Supplement 1 provides a breakdown by disposition. When looking at patient, ED facility, and ED visit characteristics, an admission was associated with lower likelihood of 30-day ED revisits (hazard ratio [HR], 0.75; 95% CI, 0.72-0.78) and increased likelihood of 30-day mortality (HR, 4.87; 95% CI, 4.36-5.45). eTables 9 and 10 in Supplement 1 show data for other modeling characteristics.

## Discussion

In this novel cohort study of veterans with dementia, we found that ED admission (vs discharge) disposition was the factor associated with the largest RR for subsequent days not at home after an index ED visit. Admitted patients spent a mean of 14.1 additional days not at home beyond the initial episode of care, highlighting that downstream health care use extends well beyond the immediate hospitalization. However, both admitted and discharged patients experienced substantial days not at home after an ED visit, and patient-level factors such as unhoused status, unmarried status, frailty, and depression were associated with poorer outcomes.

Our findings have important implications for the use of home time as a patient-centered measure of ED quality. While home time captures meaningful aspects of post-ED trajectories, it is also influenced by early care decisions, particularly admission. Among admitted patients, a large block of days not at home is directly attributable to their index hospitalization, raising questions about how best to interpret home time across different ED dispositions. Some researchers have proposed refining home time measures by weighting days differently based on care setting,^14,31^ and a similar approach could be used to weight days associated with an index admission vs subsequent health care use. However, because home time is intended as a patient-centered metric, further research is needed to understand how people with dementia and their care partners might perceive these distinctions.^32^ Alternatively, admitted and discharged patients may need to be analyzed separately, or at a minimum, disposition should be accounted for in modeling and interpretation. Additionally, while not intrinsically consistent with a high-quality day at home, postacute care can improve downstream outcomes for some patients, including reductions in ED visits^33,34^; future work should also consider how to account for the impact of these days on subsequent quality of life. These methodological concerns suggest that solutions need to be personalized to individuals, their priorities, and the ED intervention.^19^

We observed a distinct subset of patients—predominantly (but not exclusively) admitted—who had more than 100 days not at home. This likely reflects the fact that patients with dementia have high rates of transition from the hospital to postacute nursing facilities, often followed by a functional decline leading to long-term care institutionalization (these patients would have been censored in our study).^35,36^ For some patients who are able to be discharged to home after an extended SNF stay, including the 310 individuals in this study’s cohort who accrued days at home after being censored, this phenomenon may reflect the Medicare policy of covering up to 100 days in SNFs, with patients possibly returning home as coverage ends.^37^ Future research should explore these patterns in more depth and identify subgroups at risk for extended time away from home or long-term institutionalization.

Several patient-level factors were associated with increased time away from home. These included greater frailty, older age, depression, and marital status. Marital status was especially notable, as it serves as a proxy for care partner support, and other studies have similarly found that social risks like not having a partner were associated with a greater number of days not at home.^38^ Frailty has similarly been shown to be associated with increased health care use for veterans in other contexts as well.^5,39^ While these factors likely influence the initial disposition decision (and, therefore, home time), we observed similar results among patients who were discharged. We also note that associations with these characteristics remained similar when looking at different outcome durations of 90 or 365 days. Overall, this suggests that these factors remain important regardless of disposition or outcome duration. These populations may respond differently to ED care aimed at increasing home time and may require tailored interventions to ensure they can equitably benefit from future innovations.

In addition, when looking at discharge diagnoses, only ED visits for chronic conditions were associated with more days not at home. This is consistent with prior work showing higher readmission rates following ED visits related to chronic disease.^40^ However, in sensitivity analyses using patient-dictated chief concerns, several presentations common among people with dementia—such as behavioral or psychiatric issues, nonspecific concerns, and geriatric syndromes—were associated with more days not at home; notably, nonspecific concerns have been previously linked to longer hospital stays and higher mortality.^3,8^ These complex presentations may lack clear triage and diagnostic pathways and require greater resource coordination.^41,42,43^ Our findings suggest that chief concerns may better identify patients at risk for increased time away from home, though further standardization and validation for use in performance measures are required.^44,45^

### Limitations

This study has several limitations. First, patients aged 65 to 66 years at the time of their ED visit may not have had 2 years of Medicare data available for dementia diagnosis confirmation. As a result, some people with dementia may have been inadvertently excluded if their diagnosis only appeared in community care records. Second, the cohort was predominantly male, reflecting the demographics at the time of military enrollment among older adults. While this potentially limits the generalizability of the findings, some VA populations may be reasonably generalized to non-VA populations, such as male Medicare enrollees.^46^ Third, marriage status is a limited proxy variable for social and care partner support, a common problem with the use of home time measures.^47^ Fourth, we also acknowledge that people with dementia can have varying severity of dementia, which can impact levels of support needed and, ultimately, home time. VA administrative data also limit the ability to assess those factors. Fifth, we may have failed to exclude veterans with highly emergent ED visits that were not triaged as Emergency Severity Index level 1 or directly admitted to the ICU. To account for these limitations in administrative data, we excluded patients who died within 1 day of the ED visit or received urgent surgical procedures within 48 hours of admission, though these criteria are based on postbaseline events. Similarly, we did not exclude visits that were potentially very low acuity, as there is currently no validated approach for reliably identifying such visits using administrative data alone. Sixth, although the findings suggest ED disposition is associated with home time, it is difficult to determine how much to attribute subsequent days away from home to the initial disposition decision, which is a limitation in using administrative data. Seventh, it is important to note that disposition decision-making is variable.^48^ VA hospitals may have admission criteria that differ from those used in community practice, which could affect the generalizability of our findings.

## Conclusions

This cohort study found that many veterans with dementia had a substantial number of days away from home after an ED visit, and the results suggest that certain subsets of patients are more likely to have increased days away from home regardless of admission or discharge disposition at the index ED visit. Home time offers a promising, patient-centered measure to align emergency care with patients’ and care partners’ goals and preferences to remain at home. However, refining its application—particularly in accounting for index hospitalizations and long-term care transitions—is critical to accurately capturing quality of care and long-term well-being.

## Notes

Supplement 1. **eTable 1.** *ICD-9* and *ICD-10* Codes for a Dementia Diagnosis **eTable 2.** Variables and Specifications **eTable 3.** Chief Concern Categories **eTable 4.** Descriptive Counts and Summary of Days Away From Home (180 Days) **eFigure 1.** Association Between Baseline Characteristics and Days Away From Home (180 Days), Admitted Patients Only (n = 20 443) **eFigure 2.** Association Between Baseline Characteristics and Days Away From Home (180 Days), Discharged Patients Only (n = 31 264) **eTable 5.** Association Between Baseline Characteristics and Nonhome Time (180 Days), Sensitivity Analysis Including Priority Group and Prior Hospitalizations **eTable 6.** Association Between Baseline Characteristics and Days Away From Home in the Total Cohort Over 90, 180, and 365 Days **eTable 7.** Model Output Using Effect Coding for Chief Concern Category (180 Days), Total Cohort **eTable 8.** Observed Cumulative Incidence of 30-Day ED Revisits and 30-Day Mortality, Total and by Disposition **eTable 9.** Association Between Baseline Characteristics and 30-Day ED Revisits in the Total Cohort, Admitted, and Discharged **eTable 10.** Association Between Baseline Characteristics and 30-Day Mortality in the Total Cohort, Admitted, and Discharged
eReferences

Supplement 2.
Data Sharing Statement
