# Supplementary material for: Home Time Following Emergency Department Visits Among People With Dementia
Source: JAMA Netw Open. 2025 Dec 29;8(12):e2549154. doi: 10.1001/jamanetworkopen.2025.49154 (PMC12750257; doi:10.1001/jamanetworkopen.2025.49154)
Supplement: Supplement 1. — eTable 1. ICD-9 and ICD-10 Codes for a Dementia Diagnosis eTable 2. Variables and Specifications eTable 3. Chief Concern Categories eTable 4. Descriptive Counts and Summary of Days Away From Home (180 Days) eFigure 1. Association Between Baseline Characteristics and Days Away From Home (180 Days), Admitted Patients Only (n = 20 443) eFigure 2. Association Between Baseline Characteristics and Days Away From Home (180 Days), Discharged Patients Only (n = 31 264) eTable 5. Association Between Baseline Characteristics and Nonhome Time (180 Days), Sensitivity Analysis Including Priority Group and Prior Hospitalizations eTable 6. Association Between Baseline Characteristics and Days Away From Home in the Total Cohort Over 90, 180, and 365 Days eTable 7. Model Output Using Effect Coding for Chief Concern Category (180 Days), Total Cohort eTable 8. Observed Cumulative Incidence of 30-Day ED Revisits and 30-Day Mortality, Total and by Disposition eTable 9. Association Between Baseline Characteristics and 30-Day ED Revisits in the Total Cohort, Admitted, and Discharged eTable 10. Association Between Baseline Characteristics and 30-Day Mortality in the Total Cohort, Admitted, and Discharged eReferences [file jamanetwopen-e2549154-s001.pdf]

Supplementary Online Content

Seidenfeld J, Zepel L, Smith VA, et al. Home time following emergency department visits among people with dementia. *JAMA Netw Open*. 2025;8(12):e2549154.
doi:10.1001/jamanetworkopen.2025.49154

eTable 1. *ICD-9* and *ICD-10* Codes for a Dementia Diagnosis

eTable 2. Variables and Specifications

eTable 3. Chief Concern Categories

eTable 4. Descriptive Counts and Summary of Days Away From Home (180 Days)

eFigure 1. Association Between Baseline Characteristics and Days Away From Home (180 Days), Admitted Patients Only (n = 20 443)

eFigure 2. Association Between Baseline Characteristics and Days Away From Home (180 Days), Discharged Patients Only (n = 31 264)

eTable 5. Association Between Baseline Characteristics and Nonhome Time (180 Days), Sensitivity Analysis Including Priority Group and Prior Hospitalizations

eTable 6. Association Between Baseline Characteristics and Days Away From Home in the Total Cohort Over 90, 180, and 365 Days

eTable 7. Model Output Using Effect Coding for Chief Concern Category (180 Days), Total Cohort

eTable 8. Observed Cumulative Incidence of 30-Day ED Revisits and 30-Day Mortality, Total and by Disposition

eTable 9. Association Between Baseline Characteristics and 30-Day ED Revisits in the Total Cohort, Admitted, and Discharged

eTable 10. Association Between Baseline Characteristics and 30-Day Mortality in the Total Cohort, Admitted, and Discharged

eReferences

This supplementary material has been provided by the authors to give readers additional information about their work.

© 2025 Seidenfeld J, et al. *JAMA Network Open*.

eTable 1: *ICD-9* and *ICD-10* codes for a dementia diagnosis

ICD codes are drawn from the VA Office of Geriatrics and Extended Care. (Reference: Geriatrics and Extended Care. Fiscal year 2023 VHA Dementia ICD codes list. U.S. Department of Veterans Affairs. Accessed April 9, 2024.)

ICD-9 Code	ICD-9 Description
046.11	Variant Creutzfeldt-Jakob Disease
046.19	Other And Unspecified Creutzfeldt-Jakob Disease
046.3	Progressive Multifocal Leukoencephalopathy
046.71	Gerstmann-Straussler-Scheinker Syndrome
046.79	Other And Unspecified Prion Disease of Central Nervous System
046.9	Unspecified Slow Virus Infection of Central Nervous System
290.0	Senile Dementia, Uncomplicated
290.10	Presenile Dementia, Uncomplicated
290.11	Presenile Dementia with Delirium
290.12	Presenile Dementia with Delusional Features
290.13	Presenile Dementia with Depressive Features
290.20	Senile Dementia with Delusional Features
290.21	Senile Dementia with Depressive Features
290.3	Senile Dementia with Delirium
290.40	Vascular Dementia, Uncomplicated
290.41	Vascular Dementia, With Delirium
290.42	Vascular Dementia, With Delusions
290.43	Vascular Dementia, With Depressed Mood
291.1	Alcohol-Induced Persisting Amnestic Disorder
291.2	Alcohol-Induced Persisting Dementia
292.82	Drug-Induced Persisting Dementia
294.1	Dementia In Conditions Classified Elsewhere
294.10	Dementia In Conditions Classified Elsewhere Without Behavioral Disturbance
294.11	Dementia In Conditions Classified Elsewhere With Behavioral Disturbance
294.20	Dementia, Unspecified, Without Behavioral Disturbance
294.21	Dementia, Unspecified, With Behavioral Disturbance
331.0	Alzheimer's Disease
331.11	Pick's Disease
331.19	Other Frontotemporal Dementia
331.82	Dementia With Lewy Bodies
294.11/042 .0	Dementia In Conditions Classified Elsewhere With Behavioral Disturbance/HIV With Specified Infections
294.10/042.0	Dementia In Conditions Classified Elsewhere Without Behavioral Disturbance/HIV With Specified Infections
294.11/331.5	Dementia In Conditions Classified Elsewhere With Behavioral Disturbance/Idiopathic Normal Pressure Hydrocephalus (Inph)
294.10/331.5	Dementia In Conditions Classified Elsewhere Without Behavioral Disturbance/Idiopathic Normal Pressure Hydrocephalus (Inph)
294.11/332.0	Dementia In Conditions Classified Elsewhere With Behavioral Disturbance/Parkinson's Disease
294.10/332.0	Dementia In Conditions Classified Elsewhere Without Behavioral Disturbance/Parkinson's Disease
294.11/333.4	Dementia In Conditions Classified Elsewhere With Behavioral Disturbance/Huntington's Chorea
294.10/333.4	Dementia In Conditions Classified Elsewhere Without Behavioral Disturbance/Huntington's Chorea
046.11	Variant Creutzfeldt-Jakob Disease
046.19	Other And Unspecified Creutzfeldt-Jakob Disease
046.3	Progressive Multifocal Leukoencephalopathy
046.71	Gerstmann-Straussler-Scheinker Syndrome
046.79	Other And Unspecified Prion Disease Of Central Nervous System
046.9	Unspecified Slow Virus Infection Of Central Nervous System
290.0	Senile Dementia, Uncomplicated
290.10	Presenile Dementia, Uncomplicated
290.11	Presenile Dementia With Delirium

© 2025 Seidenfeld J, et al. *JAMA Network Open*.

290.12	Presenile Dementia With Delusional Features
290.13	Presenile Dementia With Depressive Features
290.20	Senile Dementia With Delusional Features
290.21	Senile Dementia With Depressive Features
290.3	Senile Dementia With Delirium
290.40	Vascular Dementia, Uncomplicated
290.41	Vascular Dementia, With Delirium
290.42	Vascular Dementia, With Delusions
290.43	Vascular Dementia, With Depressed Mood
291.1	Alcohol-Induced Persisting Amnestic Disorder
291.2	Alcohol-Induced Persisting Dementia
292.82	Drug-Induced Persisting Dementia
294.1	Dementia In Conditions Classified Elsewhere
294.10	Dementia In Conditions Classified Elsewhere Without Behavioral Disturbance
294.11	Dementia In Conditions Classified Elsewhere With Behavioral Disturbance
294.20	Dementia, Unspecified, Without Behavioral Disturbance
294.21	Dementia, Unspecified, With Behavioral Disturbance
331.0	Alzheimer's Disease
331.11	Pick's Disease
331.19	Other Frontotemporal Dementia
331.82	Dementia With Lewy Bodies
294.11/042 .0	Dementia In Conditions Classified Elsewhere With Behavioral Disturbance/HIV with Specified Infections
294.10/042.0	Dementia In Conditions Classified Elsewhere Without Behavioral Disturbance/HIV with Specified Infections
294.11/331.5	Dementia In Conditions Classified Elsewhere With Behavioral Disturbance/Idiopathic Normal Pressure Hydrocephalus
294.10/331.5	Dementia In Conditions Classified Elsewhere Without Behavioral Disturbance/Idiopathic Normal Pressure Hydrocephalus
294.11/332.0	Dementia In Conditions Classified Elsewhere With Behavioral Disturbance/Parkinson's Disease
294.10/332.0	Dementia In Conditions Classified Elsewhere Without Behavioral Disturbance/Parkinson's Disease
294.11/333.4	Dementia In Conditions Classified Elsewhere With Behavioral Disturbance/Huntington's Chorea
294.10/333.4	Dementia In Conditions Classified Elsewhere Without Behavioral Disturbance/Huntington's Chorea
**ICD-10 Code**	**ICD-10 Description**
A81.00	Creutzfeldt-Jakob Disease, Unspecified
A81.01	Variant Creutzfeldt-Jakob Disease
A81.09	Other Creutzfeldt-Jakob Disease
A81.2	Progressive Multifocal Leukoencephalopathy
A81.82	Gerstmann-Straussler-Scheinker Syndrome
F01.50	Vascular Dementia Without Behavioral Disturbance, Psychotic Disturbance, Mood Disturbance, And Anxiety
F01.51	Vascular Dementia, Unspecified Severity, With Behavioral Disturbance
F01.511	Vascular Dementia, Unspecified Severity, With Agitation
F01.518	Vascular Dementia, Unspecified Severity, With Other Behavioral Disturbance
F01.52	Vascular Dementia, Unspecified Severity, With Psychotic Disturbance
F01.53	Vascular Dementia, Unspecified Severity, With Mood Disturbance
F01.54	Vascular Dementia, Unspecified Severity, With Anxiety
F01.A0	Vascular Dementia, Mild, Without Behavioral Disturbance, Psychotic Disturbance, Mood Disturbance, And Anxiety
F01.A11	Vascular Dementia, Mild, With Agitation
F01.A18	Vascular Dementia, Mild, With Other Behavioral Disturbance
F01.A2	Vascular Dementia, Mild, With Psychotic Disturbance
F01.A3	Vascular Dementia, Mild, With Mood Disturbance
F01.A4	Vascular Dementia, Mild, With Anxiety
F01.B0	Vascular Dementia, Moderate, Without Behavioral Disturbance, Psychotic Disturbance, Mood Disturbance, And Anxiety
F01.B11	Vascular Dementia, Moderate, With Agitation
F01.B18	Vascular Dementia, Moderate, With Other Behavioral Disturbance
F01.B2	Vascular Dementia, Moderate, With Psychotic Disturbance
F01.B3	Vascular Dementia, Moderate, With Mood Disturbance

© 2025 Seidenfeld J, et al. *JAMA Network Open*.

F01.B4	Vascular Dementia, Moderate, With Anxiety
F01.C0	Vascular Dementia, Severe, Without Behavioral Disturbance, Psychotic Disturbance, Mood Disturbance, And Anxiety
F01.C11	Vascular Dementia, Severe, With Agitation
F01.C18	Vascular Dementia, Severe, With Other Behavioral Disturbance
F01.C2	Vascular Dementia, Severe, With Psychotic Disturbance
F01.C3	Vascular Dementia, Severe, With Mood Disturbance
F01.C4	Vascular Dementia, Severe, With Anxiety
F02.80	Dementia In Other Diseases Classified Elsewhere Without Behavioral Disturbance, Psychotic Disturbance, Mood Disturbance, And Anxiety
F02.81	Dementia In Other Diseases Classified Elsewhere With Behavioral Disturbance
F02.811	Dementia In Other Diseases Classified Elsewhere, Unspecified Severity, With Agitation
F02.818	Dementia In Other Diseases Classified Elsewhere, Unspecified Severity, With Other Behavioral Disturbance
F02.82	Dementia In Other Diseases Classified Elsewhere, Unspecified Severity, With Psychotic Disturbance
F02.83	Dementia In Other Diseases Classified Elsewhere, Unspecified Severity, With Mood Disturbance
F02.84	Dementia In Other Diseases Classified Elsewhere, Unspecified Severity, With Anxiety
F02.A0	Dementia In Other Diseases Classified Elsewhere, Mild, Without Behavioral Disturbance, Psychotic Disturbance, Mood Disturbance, And Anxiety
F02.A11	Dementia In Other Diseases Classified Elsewhere, Mild, With Agitation
F02.A18	Dementia In Other Diseases Classified Elsewhere, Mild, With Other Behavioral Disturbance
F02.A2	Dementia In Other Diseases Classified Elsewhere, Mild, With Psychotic Disturbance
F02.A3	Dementia In Other Diseases Classified Elsewhere, Mild, With Mood Disturbance
F02.A4	Dementia In Other Diseases Classified Elsewhere, Mild, With Anxiety
F02.B0	Dementia In Other Diseases Classified Elsewhere, Moderate, Without Behavioral Disturbance, Psychotic Disturbance, Mood Disturbance, And Anxiety
F02.B11	Dementia In Other Diseases Classified Elsewhere, Moderate, With Agitation
F02.B18	Dementia In Other Diseases Classified Elsewhere, Moderate, With Other Behavioral Disturbance
F02.B2	Dementia In Other Diseases Classified Elsewhere, Moderate, With Psychotic Disturbance
F02.B3	Dementia In Other Diseases Classified Elsewhere, Moderate, With Mood Disturbance
F02.B4	Dementia In Other Diseases Classified Elsewhere, Moderate, With Anxiety
F02.C0	Dementia In Other Diseases Classified Elsewhere, Severe, Without Behavioral Disturbance, Psychotic Disturbance, Mood Disturbance, And Anxiety
F02.C11	Dementia In Other Diseases Classified Elsewhere, Severe, With Agitation
F02.C18	Dementia In Other Diseases Classified Elsewhere, Severe, With Other Behavioral Disturbance
F02.C2	Dementia In Other Diseases Classified Elsewhere, Severe, With Psychotic Disturbance
F02.C3	Dementia In Other Diseases Classified Elsewhere, Severe, With Mood Disturbance
F02.C4	Dementia In Other Diseases Classified Elsewhere, Severe, With Anxiety
F03.90	Unspecified Dementia Without Behavioral Disturbance, Psychotic Disturbance, Mood Disturbance, And Anxiety
F03.91	Unspecified Dementia With Behavioral Disturbance
F03.911	Unspecified Dementia, Unspecified Severity, With Agitation
F03.918	Unspecified Dementia, Unspecified Severity, With Other Behavioral Disturbance
F03.92	Unspecified Dementia, Unspecified Severity, With Psychotic Disturbance
F03.93	Unspecified Dementia, Unspecified Severity, With Mood Disturbance
F03.94	Unspecified Dementia, Unspecified Severity, With Anxiety
F03.A0	Unspecified Dementia, Mild, Without Behavioral Disturbance, Psychotic Disturbance, Mood Disturbance, And Anxiety
F03.A11	Unspecified Dementia, Mild, With Agitation
F03.A18	Unspecified Dementia, Mild, With Other Behavioral Disturbance
F03.A2	Unspecified Dementia, Mild, With Psychotic Disturbance
F03.A3	Unspecified Dementia, Mild, With Mood Disturbance
F03.A4	Unspecified Dementia, Mild, With Anxiety
F03.B0	Unspecified Dementia, Moderate, Without Behavioral Disturbance, Psychotic Disturbance, Mood Disturbance, And Anxiety
F03.B11	Unspecified Dementia, Moderate, With Agitation
F03.B18	Unspecified Dementia, Moderate, With Other Behavioral Disturbance
F03.B2	Unspecified Dementia, Moderate, With Psychotic Disturbance
F03.B3	Unspecified Dementia, Moderate, With Mood Disturbance
F03.B4	Unspecified Dementia, Moderate, With Anxiety

© 2025 Seidenfeld J, et al. *JAMA Network Open*.

F03.C0	Unspecified Dementia, Severe, Without Behavioral Disturbance, Psychotic Disturbance, Mood Disturbance, And Anxiety
F03.C11	Unspecified Dementia, Severe, With Agitation
F03.C18	Unspecified Dementia, Severe, With Other Behavioral Disturbance
F03.C2	Unspecified Dementia, Severe, With Psychotic Disturbance
F03.C3	Unspecified Dementia, Severe, With Mood Disturbance
F03.C4	Unspecified Dementia, Severe, With Anxiety
F10.27	Alcohol Dependence With Alcohol-Induced Persisting Dementia
F10.97	Alcohol Use, Unspecified With Alcohol-Induced Persisting Dementia
F13.27	Sedative, Hypnotic Or Anxiolytic Dependence With Sedative, Hypnotic Or Anxiolytic-Induced Persisting Dementia
F13.97	Sedative, Hypnotic Or Anxiolytic Use, Unspecified With Sedative, Hypnotic Or Anxiolytic-Induced Persisting Dementia
F18.17	Inhalant Abuse With Inhalant-Induced Dementia
F18.27	Inhalant Dependence With Inhalant-Induced Dementia
F18.97	Inhalant Use, Unspecified With Inhalant-Induced Persisting Dementia
F19.17	Other Psychoactive Substance Abuse With Psychoactive Substance-Induced Persisting Dementia
F19.27	Other Psychoactive Substance Dependence With Psychoactive Substance-Induced Persisting Dementia
F19.97	Other Psychoactive Substance Use, Unspecified With Psychoactive Substance-Induced Persisting Dementia
G30.0	Alzheimer's Disease With Early Onset
G30.1	Alzheimer's Disease With Late Onset
G30.8	Other Alzheimer's Disease
G30.9	Alzheimer's Disease, Unspecified
G31.01	Pick's Disease
G31.09	Other Frontotemporal Dementia
G31.83	Dementia With Lewy Bodies
F02.80/B20.	Dementia In Other Diseases Classified Elsewhere Without Behavioral Disturbance/Human Immunodeficiency Virus [HIV] Disease
F02.81/B20.	Dementia In Other Diseases Classified Elsewhere With Behavioral Disturbance/Human Immunodeficiency Virus [HIV] Disease
F02.80/G10	Dementia In Other Diseases Classified Elsewhere Without Behavioral Disturbance/Huntington's Disease
F02.81/G10	Dementia In Other Diseases Classified Elsewhere With Behavioral Disturbance/Huntington's Disease
F02.80/G20	Dementia In Other Diseases Classified Elsewhere Without Behavioral Disturbance/Parkinson's Disease
F02.81/G20	Dementia In Other Diseases Classified Elsewhere With Behavioral Disturbance/Parkinson's Disease
F02.80/G91.2	Dementia In Other Diseases Classified Elsewhere Without Behavioral Disturbance/Idiopathic Normal Pressure Hydrocephalus
F02.81/G91.2	Dementia In Other Diseases Classified Elsewhere With Behavioral Disturbance/Idiopathic Normal Pressure Hydrocephalus

The following ICD codes from the VA Geriatrics and Extended Care list were removed as conditions that do not clearly include a clinical presentation of a progressive, persistent neurocognitive disorder with dementia.

ICD-10, removed:
- A81.89 Other Atypical Virus Infections of Central Nervous System
- A81.9 Atypical Virus Infection of Central Nervous System, Unspecified
- G23.1 Progressive Supranuclear Ophthalmoplegia [Steele-Richardson-Olszewski]
- G90.3 Multi-System Degeneration of the Autonomic Nervous System

ICD-9 removed:
- 046.9 Unspecified Slow Virus Infection of Central Nervous System
- 291.1 Alcohol-Induced Persisting Amnestic Disorder

© 2025 Seidenfeld J, et al. *JAMA Network Open*.

eTable 2: Variables and specifications

Variable	Specification
**Baseline variable specifications**	
JEN Frailty Index	A claims-based measure (range 0–13) of frailty derived from 13 diagnostic categories, with higher scores indicating greater risk of institutional and home-based service use. Although proprietary, it is based on standard clinical and claims data and performs comparably to other claims-based frailty measures.[1]
Service connection	Based on illness or injury related to military service and impacts health care benefits, co-payment rates, and disability compensation, with 0% being the least disabling and 100% as the most disabling.
Area Deprivation Index	The Neighborhood Atlas area deprivation index (ADI) is a composite measure of socioeconomic risk in single percentiles based on residential location based on residential location. Similar to prior methods, we elected to analyze ADI by quartile rankings.[2,3]
Facility complexity	The Facility Complexity Model uses clinical and administrative data to categorize facilities based on patient population, clinical services complexity, workload and programs (such as teaching, research, and complex clinical programs). Similar to prior work, we elected to compare the highest complexity designation 1a (most complex) to all other designations.[4]
Depression	Known depression was defined as having a documented diagnosis of depression within the past year using ICD-9 and ICD-10 codes. Likely depression was defined as a PHQ-2 score of ≥3 documented within the past 15 months. No depression was defined as having a documented PHQ-2 score of ≤2 within the past 15 months and no documented diagnosis of depression. Missing was defined as missing a PHQ-2 score and no documented diagnosis of depression.
Discharge diagnosis	These groups were modeled after prior VA based studies to identify older patients at higher risk for adverse outcomes after an ED visit.[5] They comprise of: 1) injury/musculoskeletal (MSK), 2) chronic conditions (based on the Chronic Condition Indicator (CCI) from the Healthcare Cost and Utilization Project[6]), 3) infection, 4) symptom based (non-MSK), and 5) an unclassified group to represent all other visits.
Chief concerns	As a sensitivity analysis to test the use of patient-dictated chief concerns as an alternative option to identify clinically meaningful subgroups, we used an NLP program based on the clinical Text Analysis and Knowledge Extraction System (cTAKES) applied to the original text of ED chief concerns.[7-9] A chief concern text could be mapped to zero, one, or multiple Concept Unique Identifiers (CUI) in the Unified Medical Language System (UMLS) Metathesaurus.[10] We selected all CUI with 100 or more index ED visits, and organized them into similar clinical categories to match the discharge diagnosis categories, with the addition of 1) geriatric syndromes and non-specific concerns and 2) predominantly psychiatric or behavioral health concerns. Please refer to Supplement eTable 3 for the selected categorization.
VA Priority Group	VA Enrollment Priority Groups reflect a Veteran's military service history, service-connected disability, income, Medicaid eligibility, and other VA benefits. Priority Group 1 includes Veterans with service-connected disabilities, while Priority Group 5 includes those with low income. Veterans in the highest priority groups (1–6) do not have copayments for VA care.
**Outcome variable specifications**	
For days assigned both home and non-home settings in the Residential History File[11]	We prioritized in the following order:<br>1) if there was any hospice (home) setting and a non-hospice ED or inpatient setting on the same day, this was considered a day not at home with the assumption that it is a lower quality of life day;<br>2) if there was any hospice (home) setting and any other type of non-home setting on the same day, this was considered a day at home as hospice-level care was presumably provided;<br>3) for all other days with both an assigned home and non-home setting, we counted it as a day not at home.

© 2025 Seidenfeld J, et al. *JAMA Network Open*.

<u>eTable 3:</u> Chief concern categories

Concept name	Number of visits
**Psychiatric**	
Mental health	458
Feeling suicidal (finding)	405
Referral type - Psychiatric	163
Anxiety	118
Mental Depression	111
Ethanol	108
Hallucinations	140
**Non-Specific Concerns and Geriatric Syndromes**	
Falls	4444
Abnormal mental state	1496
Dizziness	1171
Confusion	982
Mental state	493
Syncope	484
Dementia	477
Generalized muscle weakness	214
Lethargy	205
Fatigue	132
Lightheadedness	104
**Infection**	
Fever	883
Urinary tract infection	782
Congestion	396
Pneumonia	288
Common Cold	241
Productive Cough	183
Sore Throat	174
Upper Respiratory Infections	146
Dysuria	143
Influenza	139
**MSK/Trauma**	
Back Pain	889
Low Back Pain	356
Leg	290
Pain in lower limb	268
Hip joint pain	246
Neck Pain	220
Structure of right lower leg	197
Structure of left lower leg	187
Structure of right foot	187
Structure of left foot	183
Shoulder Pain	182
Knee pain	180
Examination of knee joint	168
Examination of shoulder(s)	156
Left upper arm structure	140
Pain of left hip joint	138
left shoulder joint pain	137
Pain in right hip joint	136
Toes	133
Foot pain	127
Pain of right knee joint	122
Left lower extremity	120
Pain of left knee joint	117

© 2025 Seidenfeld J, et al. *JAMA Network Open*.

Upper arm	115
Structure of left hand	114
Structure of right hand	111
Hallux structure	108
right shoulder joint pain	104
**Non-MSK symptoms**	
**Abdominal**	
Abdominal Pain	1218
Vomiting	898
Diarrhea	774
Nausea	710
Adverse Event Associated with the Gastrointestinal System	570
Constipation	375
Abdomen	175
Rectal hemorrhage	158
Blood in stool	153
Inguinal pain	118
Nausea and vomiting	112
Dehydration	174
Hernia	100
**Cardiac**	
Chest Pain	2088
Tachycardia	180
Heart	145
Chest	133
Hypotension	782
Hypertensive disease	558
Blood Pressure	213
Heart failure	193
Cardiovascular system	155
Atrial Fibrillation	115
**Respiratory**	
Dyspnea	2804
Pulmonary congestion	140
Wheezing	107
COPD	133
**Neurologic**	
Headache	508
Seizures	275
Tremor	108
Cerebrovascular accident	303
Speech	114
**Urinary**	
Hematuria	691
Urinary Retention, CTCAE	129
Increased frequency of micturition	103
**Abnormal lab**	
Hyperglycemia	299
Laboratory test finding	195
Hypoglycemia	122
**Head and Neck**	
Head	198
Eye	176
Ear structure	152
Left eye structure	142
Right eye	114
Nose	107
Neck	106

© 2025 Seidenfeld J, et al. *JAMA Network Open*.

Epistaxis	151
Pharyngeal structure	122
**Skin**	
Exanthema	391
Sore skin	140
Sore to touch	156
**Other**	
Pharmaceutical Preparations	881
Missing	10521
Problem	503
Positive Finding	319
Positive	319
Symptoms	268
Abnormal	183
Unable	136
Multiple symptoms	114
Today	113
In care (finding)	102

© 2025 Seidenfeld J, et al. *JAMA Network Open*.

eTable 4: Descriptive counts and summary of days away from home (180 days)

	Minimum	5th Pctl	25th Pctl	50th Pctl	75th Pctl	95th Pctl	Maximum	Mean	Std Dev
Total cohort (**N=51,707)**	0	0	1	6	25	107	180	21.74	34.53
Discharged **(N=31,264)**	0	0	0	2	11	84	180	13.6	27.06
Admitted **(N=20,443)**	0	2	5	15	48	117	180	34.18	40.45
Admitted, index inpatient hospital days	0	1	2	4	7	22	180	7.03	12.46
Admitted, index episode of care	0	1	2	5	20	106	180	20.13	32.89
Admitted, days not in index episode of care	0	0	0	1	13	85	177	14.05	27.15

Abbreviations: Pctl=percentile; Std Dev=standard deviation

© 2025 Seidenfeld J, et al. *JAMA Network Open*.

eFigure 1: Association between baseline characteristics and days away from home (180 days), admitted patients only (n=20,443)*

Characteristic	RR (95% CI)
ED visit discharge diagnosis category, injury and MSK (ref: Non-MSK)	1.32 (1.23, 1.42)
Unhoused (ref: no)	1.29 (1.18, 1.40)
Marital status unknown (ref: married)	1.26 (1.05, 1.51)
Never married (ref: married)	1.22 (1.14, 1.30)
Missing data to determine depression (ref: No depression)	1.16 (1.11, 1.22)
Unknown or missing race (ref: White)	1.14 (1.04, 1.25)
Missing area deprivation index decile (ref: 76-100)	1.12 (1.00, 1.26)
Divorced, separated or widowed (ref: married)	1.12 (1.09, 1.16)
Missing Jen Frailty Index (ref: 0-3)	1.10 (1.00, 1.21)
ED visit discharge diagnosis category, CCIR (ref: Non-MSK)	1.09 (1.04, 1.14)
Missing rurality (ref: urban)	1.08 (0.89, 1.31)
Known or likely depression (ref: No depression)	1.06 (1.02, 1.10)
Unknown ethnicity (ref: Non-Hispanic)	1.05 (0.94, 1.18)
Native Hawaiian or Pacific Islander (ref: White)	1.05 (0.87, 1.26)
Age, per 5 years	1.04 (1.03, 1.05)
ED visit discharge diagnosis category, other (ref: Non-MSK)	1.04 (0.99, 1.08)
Service connected, 100% (ref: no)	1.03 (0.98, 1.09)
Annual ED volume, 10000<-20000 (ref: 20000<-30000)	1.03 (0.95, 1.10)
Jen Frailty Index, 8+ (ref: 0-3)	1.02 (0.97, 1.07)
ED visit discharge diagnosis category, infection (ref: Non-MSK)	1.01 (0.96, 1.06)
Area deprivation index decile, 0-25 (ref: 76-100)	1.00 (0.95, 1.07)
Service connected, 50-99% (ref: no)	1.00 (0.96, 1.05)
Rural facility (ref: urban)	1.00 (0.86, 1.16)
Area deprivation index decile, 51-75 (ref: 76-100)	0.99 (0.95, 1.04)
Facility complexity score 1b, 1c, 2, 3 (ref: 1a)	0.99 (0.92, 1.07)
Driving distance to nearest VAMC > 40 miles (ref: <= 40 miles)	0.99 (0.91, 1.08)
Missing driving distance to nearest VAMC (ref: <=40 miles)	0.99 (0.89, 1.10)
Rural (ref: urban)	0.99 (0.94, 1.03)
American or Alaska Native (ref: White)	0.99 (0.80, 1.21)
Black or African-American (ref: White)	0.98 (0.94, 1.03)
Service connected, 0-49% (ref: no)	0.97 (0.93, 1.01)
Jen Frailty Index, 6-7 (ref: 0-3)	0.96 (0.91, 1.01)
Annual ED volume, <=10000 (ref: 20000<-30000)	0.96 (0.84, 1.10)
Annual ED volume, >30000 (ref: 20000<-30000)	0.95 (0.86, 1.05)
Area deprivation index decile, 26-50 (ref: 76-100)	0.94 (0.90, 0.99)
Multiple races (ref: White)	0.94 (0.78, 1.12)
Jen Frailty Index, 4-5 (ref: 0-3)	0.94 (0.89, 0.99)
Rural other (ref: urban)	0.86 (0.72, 1.04)
Female (ref: male)	0.80 (0.72, 0.89)
Hispanic (ref: Non-Hispanic)	0.78 (0.72, 0.84)
Asian (ref: White)	0.65 (0.51, 0.82)

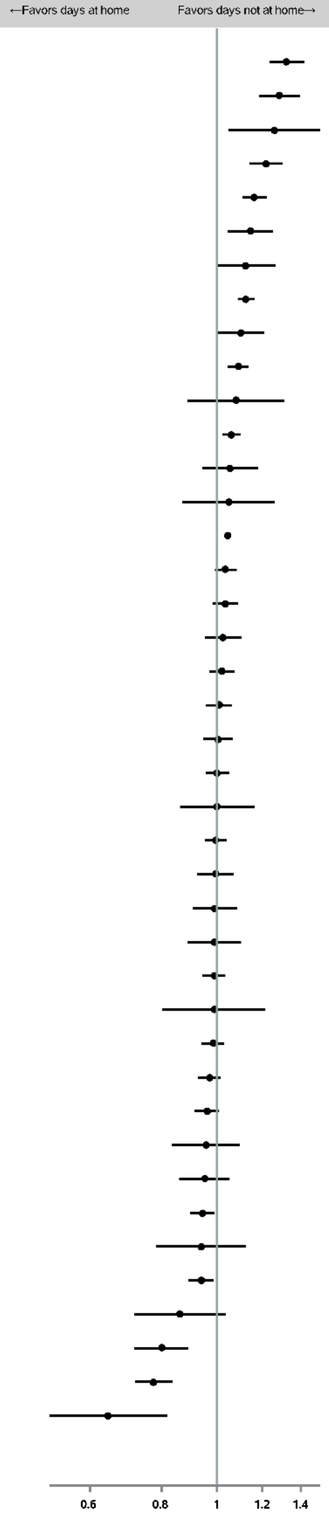

*Model adjusted for all variables and accounting for clustering within VA medical center.

© 2025 Seidenfeld J, et al. *JAMA Network Open*.

eFigure 2: Association between baseline characteristics and days away from home (180 days), discharged patients only (n=31,264)*

Characteristic	RR (95% CI)
Unhoused (ref: no)	1.66 (1.47, 1.87)
Jen Frailty Index, 8+ (ref: 0-3)	1.47 (1.36, 1.59)
Missing Jen Frailty Index (ref: 0-3)	1.34 (1.14, 1.59)
Missing rurality (ref: urban)	1.33 (0.95, 1.86)
Divorced, separated or widowed (ref: married)	1.31 (1.25, 1.38)
Marital status unknown (ref: married)	1.27 (0.95, 1.68)
Never married (ref: married)	1.26 (1.13, 1.39)
Missing data to determine depression (ref: No depression)	1.17 (1.08, 1.26)
Known or likely depression (ref: No depression)	1.16 (1.10, 1.23)
ED visit discharge diagnosis category, CCIR (ref: Non-MSK)	1.16 (1.08, 1.25)
Jen Frailty Index, 6-7 (ref: 0-3)	1.14 (1.06, 1.23)
Service connected, 100% (ref: no)	1.11 (1.03, 1.19)
Rural facility (ref: urban)	1.09 (0.94, 1.26)
American or Alaska Native (ref: White)	1.09 (0.81, 1.46)
Unknown ethnicity (ref: Non-Hispanic)	1.08 (0.89, 1.31)
Age, per 5 years	1.07 (1.05, 1.08)
Annual ED volume, 10000<-20000 (ref: 20000<-30000)	1.06 (0.98, 1.15)
Annual ED volume, <=10000 (ref: 20000<-30000)	1.04 (0.91, 1.20)
Black or African-American (ref: White)	1.03 (0.97, 1.10)
Unknown or missing race (ref: White)	1.01 (0.87, 1.18)
Missing driving distance to nearest VAMC (ref: <=40 miles)	1.01 (0.85, 1.19)
Facility complexity score 1b, 1c, 2, 3 (ref: 1a)	1.00 (0.93, 1.08)
ED visit discharge diagnosis category, other (ref: Non-MSK)	0.99 (0.92, 1.05)
Service connected, 0-49% (ref: no)	0.98 (0.91, 1.04)
Missing area deprivation index decile (ref: 76-100)	0.97 (0.81, 1.17)
ED visit discharge diagnosis category, infection (ref: Non-MSK)	0.97 (0.89, 1.06)
ED visit discharge diagnosis category, injury and MSK (ref: Non-MSK)	0.97 (0.90, 1.04)
Service connected, 50-99% (ref: no)	0.96 (0.90, 1.03)
Rural (ref: urban)	0.96 (0.90, 1.02)
Native Hawaiian or Pacific Islander (ref: White)	0.95 (0.72, 1.26)
Annual ED volume, >30000 (ref: 20000<-30000)	0.95 (0.86, 1.04)
Driving distance to nearest VAMC > 40 miles (ref: <= 40 miles)	0.94 (0.83, 1.06)
Area deprivation index decile, 0-25 (ref: 76-100)	0.93 (0.86, 1.02)
Area deprivation index decile, 26-50 (ref: 76-100)	0.93 (0.87, 1.00)
Area deprivation index decile, 51-75 (ref: 76-100)	0.93 (0.87, 0.99)
Jen Frailty Index, 4-5 (ref: 0-3)	0.92 (0.85, 0.99)
Female (ref: male)	0.91 (0.78, 1.06)
Multiple races (ref: White)	0.91 (0.69, 1.19)
Hispanic (ref: Non-Hispanic)	0.82 (0.74, 0.91)
Asian (ref: White)	0.79 (0.56, 1.11)
Rural other (ref: urban)	0.79 (0.59, 1.05)

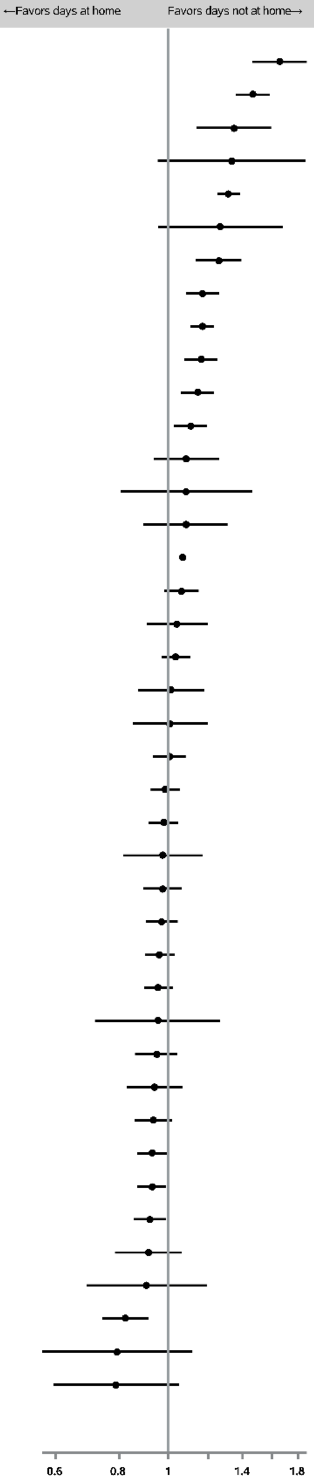

*Model adjusted for all variables and accounting for clustering within VA medical center.

© 2025 Seidenfeld J, et al. *JAMA Network Open*.

eTable 5: Association between baseline characteristics and non-home time (180 days), sensitivity analysis including priority group and prior hospitalizations

	**Total (N=51,707)**
Parameter	RR (95% CI)
Admit disposition (ref: discharge disposition)	3.15 (3.05, 3.26)
Age, per 5 years	1.07 (1.06, 1.08)
Female (ref: male)	0.88 (0.79, 0.97)
Hispanic (ref: Non-Hispanic)	0.81 (0.75, 0.87)
Unknown ethnicity (ref: Non-Hispanic)	1.07 (0.95, 1.20)
American or Alaska Native (ref: White)	1.08 (0.89, 1.31)
Asian (ref: White)	0.74 (0.59, 0.93)
Black or African-American (ref: White)	1.00 (0.96, 1.05)
Native Hawaiian or Pacific Islander (ref: White)	1.01 (0.84, 1.21)
Multiple races (ref: White)	0.90 (0.75, 1.07)
Unknown or missing race (ref: White)	1.08 (0.99, 1.19)
Rural (ref: urban)	0.97 (0.93, 1.01)
Rural other (ref: urban)	0.83 (0.69, 0.99)
Missing rurality (ref: urban)	1.22 (0.99, 1.50)
Never married (ref: married)	1.21 (1.14, 1.30)
Marital status unknown (ref: married)	1.27 (1.06, 1.52)
Divorced, separated or widowed (ref: married)	1.22 (1.18, 1.26)
Driving distance to nearest VAMC > 40 miles (ref: <= 40 miles)	0.96 (0.89, 1.05)
Missing driving distance to nearest VAMC (ref: <=40 miles)	1.01 (0.91, 1.12)
Area deprivation index decile, 0-25 (ref: 76-100)	0.97 (0.91, 1.03)
Area deprivation index decile, 26-50 (ref: 76-100)	0.95 (0.90, 0.99)
Area deprivation index decile, 51-75 (ref: 76-100)	0.96 (0.92, 1.00)
Missing area deprivation index decile (ref: 76-100)	1.05 (0.93, 1.18)
Jen Frailty Index, 4-5 (ref: 0-3)	0.92 (0.88, 0.97)
Jen Frailty Index, 6-7 (ref: 0-3)	1.06 (1.01, 1.11)
Jen Frailty Index, 8+ (ref: 0-3)	1.25 (1.19, 1.31)
Missing Jen Frailty Index (ref: 0-3)	1.24 (1.12, 1.37)
Unhoused (ref: no)	1.45 (1.34, 1.58)
Service connected, 0-49% (ref: no)	0.95 (0.88, 1.02)
Service connected, 50-99% (ref: no)	0.92 (0.87, 0.98)
Service connected, 100% (ref: no)	1.02 (0.95, 1.09)
**Low/moderate disability (groups 2, 3, 6) (ref: highly disabled [groups 1,4])**	**0.98 (0.90, 1.07)**
**Low income (group 5) (ref: highly disabled [groups 1,4])**	**0.97 (0.91, 1.02)**
**Nondisabled, co-payment required (groups 7,8) (ref: highly disabled [groups 1,4])**	**0.88 (0.83, 0.94)**
**Missing data to determine disability (ref: highly disabled [groups 1,4])**	**0.60 (0.24, 1.53)**
Known or likely depression (ref: No depression)	1.12 (1.08, 1.16)
Missing data to determine depression (ref: No depression)	1.19 (1.14, 1.25)
**Prior hospitalization (ref: No Prior hospitalization)**	**1.30 (1.25, 1.36)**
Rural facility (ref: urban)	1.06 (0.93, 1.19)
Facility complexity score 1b, 1c, 2, 3 (ref: 1a)	1.00 (0.94, 1.06)
Annual ED volume, <=10000 (ref: 20000<-30000)	1.02 (0.91, 1.14)
Annual ED volume, 10000<-20000 (ref: 20000<-30000)	1.06 (0.99, 1.13)
Annual ED volume, >30000 (ref: 20000<-30000)	0.95 (0.88, 1.04)
Discharge diagnosis, chronic conditions (ref: Non-MSK)	1.13 (1.08, 1.18)
Discharge diagnosis, injury and MSK (ref: Non-MSK)	1.03 (0.98, 1.08)
Discharge diagnosis, infection (ref: Non-MSK)	0.98 (0.93, 1.03)
Discharge diagnosis, other (ref: Non-MSK)	1.00 (0.95, 1.04)

Model adjusted for all variables in table and accounting for clustering within VA medical center.
Abbreviations: CI = Confidence Interval; ED = Emergency Department; MSK = Musculoskeletal; n = number; ref = reference; RR = rate ratio; VAMC = Veterans Affairs Medical Center.

© 2025 Seidenfeld J, et al. *JAMA Network Open*.

eTable 6: Association between baseline characteristics and days away from home in the total cohort over 90, 180, and 365 days

	**90 days**	**180 days**	**365 days**
Parameter	RR (95% CI)	*RR (95% CI)*	RR (95% CI)
Admit disposition (ref: discharge disposition)	3.97 (3.84, 4.11)	3.18 (3.08, 3.29)	2.71 (2.63, 2.79)
Age, per 5 years	1.05 (1.04, 1.06)	1.06 (1.05, 1.07)	1.06 (1.05, 1.07)
Female (ref: male)	0.91 (0.81, 1.01)	0.88 (0.79, 0.97)	0.86 (0.78, 0.94)
Hispanic (ref: Non-Hispanic)	0.83 (0.77, 0.89)	0.82 (0.77, 0.88)	0.84 (0.78, 0.90)
Unknown ethnicity (ref: Non-Hispanic)	1.06 (0.93, 1.19)	1.07 (0.95, 1.20)	1.05 (0.95, 1.17)
American or Alaska Native (ref: White)	1.09 (0.88, 1.34)	1.07 (0.88, 1.30)	1.02 (0.85, 1.22)
Asian (ref: White)	0.81 (0.64, 1.03)	0.76 (0.60, 0.95)	0.70 (0.57, 0.86)
Black or African-American (ref: White)	1.00 (0.96, 1.05)	1.01 (0.97, 1.06)	1.01 (0.97, 1.05)
Native Hawaiian or Pacific Islander (ref: White)	1.03 (0.84, 1.24)	0.99 (0.83, 1.19)	0.96 (0.81, 1.14)
Multiple races (ref: White)	0.96 (0.79, 1.15)	0.92 (0.77, 1.09)	0.91 (0.77, 1.07)
Unknown or missing race (ref: White)	1.06 (0.96, 1.17)	1.07 (0.97, 1.17)	1.05 (0.97, 1.15)
Rural (ref: urban)	0.98 (0.94, 1.03)	0.97 (0.93, 1.01)	0.97 (0.93, 1.01)
Other (ref: urban)	0.85 (0.70, 1.03)	0.83 (0.69, 0.99)	0.84 (0.71, 0.99)
Missing (ref: urban)	1.20 (0.96, 1.49)	1.20 (0.98, 1.47)	1.13 (0.94, 1.37)
Never married (ref: married)	1.23 (1.15, 1.32)	1.24 (1.16, 1.32)	1.26 (1.19, 1.34)
Marital status unknown (ref: married)	1.29 (1.06, 1.56)	1.28 (1.07, 1.53)	1.28 (1.08, 1.51)
Divorced, separated or widowed (ref: married)	1.23 (1.18, 1.27)	1.24 (1.20, 1.28)	1.24 (1.20, 1.28)
Driving distance to nearest VAMC > 40 miles (ref: <= 40 miles)	0.96 (0.88, 1.05)	0.96 (0.88, 1.04)	0.96 (0.89, 1.04)
Missing driving distance to nearest VAMC (ref: <=40 miles)	0.99 (0.88, 1.11)	1.00 (0.90, 1.12)	1.03 (0.94, 1.14)
Area deprivation index decile, 0-25 (ref: 76-100)	0.96 (0.91, 1.02)	0.96 (0.90, 1.01)	0.95 (0.90, 1.01)
Area deprivation index decile, 26-50 (ref: 76-100)	0.94 (0.89, 0.98)	0.94 (0.90, 0.98)	0.94 (0.91, 0.98)
Area deprivation index decile, 51-75 (ref: 76-100)	0.95 (0.91, 0.99)	0.96 (0.92, 1.00)	0.97 (0.93, 1.01)
Missing area deprivation index decile (ref: 76-100)	1.05 (0.93, 1.19)	1.04 (0.93, 1.17)	1.06 (0.95, 1.18)
Jen Frailty Index, 0-3 (ref >= 8)	0.79 (0.75, 0.83)	0.79 (0.75, 0.83)	0.79 (0.75, 0.82)
Jen Frailty Index, 4-5 (ref >= 8)	0.73 (0.69, 0.76)	0.73 (0.70, 0.76)	0.74 (0.71, 0.77)
Jen Frailty Index, 6-7 (ref >= 8)	0.83 (0.80, 0.87)	0.84 (0.81, 0.88)	0.84 (0.81, 0.87)
Missing Jen Frailty Index, 0-3 (ref >= 8)	1.01 (0.90, 1.12)	0.97 (0.88, 1.07)	0.95 (0.87, 1.04)
Homeless (ref: no)	1.50 (1.38, 1.63)	1.50 (1.39, 1.63)	1.45 (1.35, 1.56)
Service connected, 0-49% (ref: no)	0.98 (0.93, 1.02)	0.97 (0.93, 1.01)	0.96 (0.93, 1.00)
Service connected, 50-99% (ref: no)	0.94 (0.90, 0.99)	0.97 (0.93, 1.01)	0.97 (0.93, 1.01)
Service connected, 1009% (ref: no)	1.04 (0.99, 1.10)	1.08 (1.02, 1.13)	1.09 (1.05, 1.15)
Known or likely depression (ref: No depression)	1.14 (1.10, 1.18)	1.13 (1.09, 1.17)	1.12 (1.08, 1.16)
Missing data on depression (ref: No depression)	1.16 (1.10, 1.22)	1.17 (1.12, 1.23)	1.18 (1.13, 1.23)
Rural facility (ref: urban)	1.06 (0.94, 1.20)	1.06 (0.94, 1.20)	1.03 (0.91, 1.16)
Facility complexity score 1b, 1c, 2, 3 (ref: 1a)	0.99 (0.93, 1.05)	0.99 (0.93, 1.06)	1.00 (0.93, 1.07)
Annual ED volume, <=10000 (ref: 20000<-30000)	0.98 (0.87, 1.09)	1.01 (0.90, 1.13)	1.02 (0.91, 1.15)
Annual ED volume, 10000<-20000 (ref: 20000<-30000)	1.05 (0.99, 1.12)	1.05 (0.99, 1.12)	1.06 (0.99, 1.13)
Annual ED volume, >30000 (ref: 20000<-30000)	0.98 (0.90, 1.06)	0.95 (0.87, 1.03)	0.92 (0.84, 1.01)
Discharge diagnosis, chronic conditions (ref: Non-MSK)	1.13 (1.07, 1.18)	1.13 (1.08, 1.18)	1.11 (1.07, 1.16)
Discharge diagnosis, injury and MSK (ref: Non-MSK)	1.05 (1.00, 1.11)	1.03 (0.98, 1.08)	1.01 (0.97, 1.06)
Discharge diagnosis, infection (ref: Non-MSK)	0.97 (0.92, 1.03)	0.98 (0.93, 1.04)	0.97 (0.93, 1.02)
Discharge diagnosis, other (ref: Non-MSK)	0.99 (0.95, 1.04)	1.00 (0.96, 1.04)	1.00 (0.96, 1.04)

Abbreviations: ED = Emergency Department; MSK = Musculoskeletal; n = number; ref = reference; RR = rate ratio; VAMC = Veterans Affairs Medical Center.

© 2025 Seidenfeld J, et al. *JAMA Network Open*.

eTable 7: Model output using effect coding for chief concern category, (180 days), total cohort

Chief concern category	Unadjusted mean non-home time days	Model estimated mean non-home time days (95% CI)	Model estimated rate ratio (relative to the overall mean) (95% CI)	p-value
Psychiatric	42.12	34.62 (31.09, 38.55)	1.31 (1.19, 1.43)	<.0001
Non-specific concerns and geriatric syndromes	27.82	31.47 (30.17, 32.83)	1.19 (1.14, 1.24)	<.0001
< 100 visits in chief concern category	21.68	26.53 (25.59, 27.51)	1.00 (0.97, 1.04)	0.8887
MSK+Trauma	19.88	26.49 (25.20, 27.84)	1.00 (0.96, 1.05)	0.9701
Other	17.80	24.28 (22.07, 26.72)	0.92 (0.85, 1.00)	0.0396
Non-MSK symptoms	18.55	22.47 (21.66, 23.32)	0.85 (0.82, 0.88)	<.0001
Infection	17.06	21.78 (20.48, 23.15)	0.82 (0.78, 0.87)	<.0001

Model adjusted for patient-level and facility-level characteristics and accounting for clustering within Veterans Affairs Medical Center..
Abbreviations: CI= Confidence Interval; MSK = Musculoskeletal.

© 2025 Seidenfeld J, et al. *JAMA Network Open*.

eTable 8: Observed cumulative incidence of 30-day ED Revisits and 30-Day Mortality, total and by disposition

	**Total (N=51,707)**	**Discharged (N=31,264)**	**Admitted (N=20,443)**
30 Day ED Revisits	14274 (27.6)	9300 (29.7)	4974 (24.3)
30 Day Mortality	2065 (4.0)	1580 (7.7)	485 (1.6)

Abbreviations: ED = Emergency Department.

© 2025 Seidenfeld J, et al. *JAMA Network Open*.

eTable 9: Association between baseline characteristics and 30-day ED revisits in the total cohort, admitted, and discharged

	Total (N=51,707)	Discharged (N=31,264)	Admitted (N=20,443)
Parameter	*HR (95% CI)*	*HR (95% CI)*	*HR (95% CI)*
Admit disposition (ref: discharge disposition)	0.75 (0.72, 0.78)		
Age, per 5 years	1.02 (1.01, 1.03)	1.39 (1.31, 1.47)	1.00 (0.98, 1.02)
Female (ref: male)	0.90 (0.81, 0.99)	1.12 (0.66, 1.88)	0.74 (0.60, 0.92)
Hispanic (ref: Non-Hispanic)	0.95 (0.90, 1.00)	1.15 (0.85, 1.54)	0.90 (0.80, 1.02)
Unknown ethnicity (ref: Non-Hispanic)	0.94 (0.82, 1.07)	1.14 (0.66, 1.97)	0.83 (0.66, 1.03)
American or Alaska Native (ref: White)	1.06 (0.90, 1.26)	1.09 (0.37, 3.23)	1.04 (0.73, 1.48)
Asian (ref: White)	0.98 (0.80, 1.18)	2.00 (1.04, 3.85)	0.81 (0.53, 1.22)
Black or African-American (ref: White)	0.99 (0.95, 1.04)	0.89 (0.68, 1.16)	0.97 (0.89, 1.05)
Native Hawaiian or Pacific Islander (ref: White)	1.06 (0.86, 1.30)	0.90 (0.32, 2.49)	1.06 (0.77, 1.46)
Multiple races (ref: White)	1.01 (0.83, 1.22)	0.30 (0.04, 2.17)	1.04 (0.77, 1.41)
Unknown or missing race (ref: White)	0.94 (0.84, 1.04)	1.57 (1.00, 2.47)	0.90 (0.74, 1.09)
Rural (ref: urban)	0.98 (0.94, 1.03)	1.13 (0.87, 1.46)	0.99 (0.91, 1.08)
Other (ref: urban)	0.99 (0.82, 1.20)	1.36 (0.53, 3.47)	0.90 (0.67, 1.20)
Missing (ref: urban)	1.17 (0.95, 1.45)	0.72 (0.25, 2.03)	1.02 (0.73, 1.44)
Never married (ref: married)	1.12 (1.05, 1.19)	0.98 (0.63, 1.52)	1.01 (0.90, 1.13)
Marital status unknown (ref: married)	1.14 (0.97, 1.35)	1.56 (0.78, 3.14)	0.88 (0.64, 1.22)
Divorced, separated or widowed (ref: married)	1.10 (1.06, 1.14)	1.09 (0.91, 1.31)	1.02 (0.96, 1.09)
Driving distance to nearest VAMC > 40 miles (ref: <= 40 miles)	0.95 (0.87, 1.04)	0.76 (0.48, 1.21)	1.01 (0.88, 1.16)
Missing driving distance to nearest VAMC (ref: <=40 miles)	1.02 (0.91, 1.14)	0.98 (0.55, 1.73)	1.05 (0.88, 1.25)
Area deprivation index decile, 0-25 (ref: 76-100)	0.98 (0.93, 1.04)	0.87 (0.62, 1.22)	0.95 (0.87, 1.05)
Area deprivation index decile, 26-50 (ref: 76-100)	0.97 (0.92, 1.01)	0.91 (0.71, 1.18)	0.98 (0.90, 1.06)
Area deprivation index decile, 51-75 (ref: 76-100)	0.98 (0.93, 1.03)	1.01 (0.81, 1.26)	0.97 (0.89, 1.05)
Missing area deprivation index decile (ref: 76-100)	1.08 (0.98, 1.20)	0.84 (0.53, 1.33)	1.06 (0.87, 1.30)
Jen Frailty Index, 0-3 (ref >= 8)	0.98 (0.93, 1.04)	0.90 (0.67, 1.21)	1.05 (0.94, 1.16)
Jen Frailty Index, 4-5 (ref >= 8)	1.13 (1.07, 1.19)	1.09 (0.84, 1.42)	1.18 (1.07, 1.31)
Jen Frailty Index, 6-7 (ref >= 8)	1.39 (1.32, 1.47)	1.15 (0.88, 1.49)	1.44 (1.31, 1.59)
Missing Jen Frailty Index, 0-3 (ref >= 8)	1.14 (1.00, 1.30)	1.54 (0.96, 2.49)	1.09 (0.90, 1.31)
Homeless (ref: no)	1.54 (1.43, 1.66)	0.65 (0.35, 1.21)	1.20 (1.05, 1.36)
Service connected, 0-49% (ref: no)	0.98 (0.93, 1.03)	0.99 (0.78, 1.25)	1.06 (0.98, 1.15)
Service connected, 50-99% (ref: no)	0.94 (0.90, 0.98)	0.74 (0.54, 1.01)	0.99 (0.91, 1.08)
Service connected, 1009% (ref: no)	1.00 (0.95, 1.05)	0.83 (0.62, 1.12)	1.05 (0.95, 1.16)
Known or likely depression (ref: No depression)	1.08 (1.04, 1.12)	1.14 (0.90, 1.44)	1.01 (0.95, 1.07)
Missing data on depression (ref: No depression)	0.96 (0.92, 1.01)	1.42 (1.13, 1.77)	0.90 (0.82, 0.99)
Rural facility (ref: urban)	1.10 (1.00, 1.20)	1.28 (0.76, 2.15)	1.07 (0.85, 1.34)
Facility complexity score 1b, 1c, 2, 3 (ref: 1a)	1.04 (0.99, 1.08)	1.22 (0.93, 1.61)	0.99 (0.92, 1.07)
Annual ED volume, <=10000 (ref: 20000<-30000)	0.89 (0.78, 1.02)	1.11 (0.75, 1.64)	0.83 (0.70, 0.99)
Annual ED volume, 10000<-20000 (ref: 20000<-30000)	1.03 (0.98, 1.09)	0.99 (0.76, 1.28)	1.02 (0.94, 1.10)
Annual ED volume, >30000 (ref: 20000<-30000)	1.03 (0.98, 1.08)	1.25 (0.94, 1.66)	0.97 (0.89, 1.06)
Discharge diagnosis, chronic conditions (ref: Non-MSK)	1.04 (1.00, 1.09)	1.24 (0.97, 1.60)	1.04 (0.97, 1.11)
Discharge diagnosis, injury and MSK (ref: Non-MSK)	0.91 (0.86, 0.97)	0.80 (0.58, 1.10)	0.88 (0.75, 1.04)
Discharge diagnosis, infection (ref: Non-MSK)	0.99 (0.93, 1.06)	1.08 (0.78, 1.49)	0.97 (0.89, 1.06)
Discharge diagnosis, other (ref: Non-MSK)	0.98 (0.93, 1.02)	0.91 (0.72, 1.16)	1.05 (0.98, 1.13)

Abbreviations: ED = Emergency Department; HR = hazard ratio; MSK = Musculoskeletal; n = number; VAMC = Veterans Affairs Medical Center.

© 2025 Seidenfeld J, et al. *JAMA Network Open*.

eTable 10: Association between baseline characteristics and 30-day mortality in the total cohort, admitted, and discharged

	**Total (N=51,707)**	**Discharged (N=31,264)**	**Admitted (N=20,443)**
Parameter	HR (95% CI)	*HR (95% CI)*	HR (95% CI)
Admit disposition (ref: discharge disposition)	4.87 (4.36, 5.45)		
Age, per 5 years	1.36 (1.32, 1.40)	1.39 (1.31, 1.47)	1.35 (1.30, 1.40)
Female (ref: male)	0.72 (0.53, 0.97)	1.12 (0.66, 1.88)	0.59 (0.40, 0.87)
Hispanic (ref: Non-Hispanic)	1.13 (0.89, 1.43)	1.15 (0.85, 1.54)	1.12 (0.87, 1.43)
Unknown ethnicity (ref: Non-Hispanic)	1.08 (0.81, 1.45)	1.14 (0.66, 1.97)	1.06 (0.77, 1.47)
American or Alaska Native (ref: White)	1.04 (0.60, 1.79)	1.09 (0.37, 3.23)	1.03 (0.57, 1.86)
Asian (ref: White)	0.69 (0.37, 1.27)	2.00 (1.04, 3.85)	0.33 (0.11, 0.95)
Black or African-American (ref: White)	0.83 (0.73, 0.94)	0.89 (0.68, 1.16)	0.81 (0.71, 0.92)
Native Hawaiian or Pacific Islander (ref: White)	0.94 (0.56, 1.59)	0.90 (0.32, 2.49)	0.95 (0.52, 1.74)
Multiple races (ref: White)	0.70 (0.40, 1.21)	0.30 (0.04, 2.17)	0.81 (0.46, 1.42)
Unknown or missing race (ref: White)	1.39 (1.14, 1.69)	1.57 (1.00, 2.47)	1.35 (1.08, 1.68)
Rural (ref: urban)	1.10 (0.98, 1.24)	1.13 (0.87, 1.46)	1.09 (0.94, 1.27)
Other (ref: urban)	1.43 (0.88, 2.32)	1.36 (0.53, 3.47)	1.50 (0.96, 2.34)
Missing (ref: urban)	0.67 (0.40, 1.12)	0.72 (0.25, 2.03)	0.67 (0.38, 1.18)
Never married (ref: married)	0.95 (0.78, 1.16)	0.98 (0.63, 1.52)	0.94 (0.76, 1.16)
Marital status unknown (ref: married)	1.19 (0.69, 2.06)	1.56 (0.78, 3.14)	1.09 (0.55, 2.19)
Divorced, separated or widowed (ref: married)	0.90 (0.82, 0.98)	1.09 (0.91, 1.31)	0.84 (0.76, 0.93)
Driving distance to nearest VAMC > 40 miles (ref: <= 40 miles)	0.76 (0.57, 1.03)	0.76 (0.48, 1.21)	0.76 (0.54, 1.09)
Missing driving distance to nearest VAMC (ref: <=40 miles)	0.96 (0.71, 1.29)	0.98 (0.55, 1.73)	0.95 (0.68, 1.32)
Area deprivation index decile, 0-25 (ref: 76-100)	0.99 (0.85, 1.16)	0.87 (0.62, 1.22)	1.04 (0.88, 1.22)
Area deprivation index decile, 26-50 (ref: 76-100)	0.90 (0.79, 1.02)	0.91 (0.71, 1.18)	0.90 (0.77, 1.05)
Area deprivation index decile, 51-75 (ref: 76-100)	1.04 (0.92, 1.17)	1.01 (0.81, 1.26)	1.05 (0.93, 1.19)
Missing area deprivation index decile (ref: 76-100)	1.07 (0.81, 1.41)	0.84 (0.53, 1.33)	1.14 (0.83, 1.57)
Jen Frailty Index, 0-3 (ref >= 8)	0.93 (0.83, 1.04)	0.90 (0.67, 1.21)	0.94 (0.83, 1.07)
Jen Frailty Index, 4-5 (ref >= 8)	0.99 (0.89, 1.11)	1.09 (0.84, 1.42)	0.96 (0.84, 1.09)
Jen Frailty Index, 6-7 (ref >= 8)	0.92 (0.83, 1.03)	1.15 (0.88, 1.49)	0.86 (0.76, 0.98)
Missing Jen Frailty Index, 0-3 (ref >= 8)	1.11 (0.88, 1.38)	1.54 (0.96, 2.49)	1.02 (0.77, 1.35)
Homeless (ref: no)	0.56 (0.40, 0.80)	0.65 (0.35, 1.21)	0.54 (0.35, 0.82)
Service connected, 0-49% (ref: no)	0.94 (0.86, 1.04)	0.99 (0.78, 1.25)	0.93 (0.83, 1.05)
Service connected, 50-99% (ref: no)	0.86 (0.74, 0.99)	0.74 (0.54, 1.01)	0.90 (0.77, 1.06)
Service connected, 1009% (ref: no)	0.93 (0.81, 1.08)	0.83 (0.62, 1.12)	0.97 (0.82, 1.15)
Known or likely depression (ref: No depression)	1.10 (0.98, 1.23)	1.14 (0.90, 1.44)	1.08 (0.96, 1.21)
Missing data on depression (ref: No depression)	1.28 (1.12, 1.47)	1.42 (1.13, 1.77)	1.24 (1.06, 1.46)
Rural facility (ref: urban)	1.00 (0.70, 1.43)	1.28 (0.76, 2.15)	0.89 (0.55, 1.42)
Facility complexity score 1b, 1c, 2, 3 (ref: 1a)	1.29 (1.12, 1.48)	1.22 (0.93, 1.61)	1.31 (1.13, 1.52)
Annual ED volume, <=10000 (ref: 20000<-30000)	0.67 (0.49, 0.91)	1.11 (0.75, 1.64)	0.52 (0.35, 0.77)
Annual ED volume, 10000<-20000 (ref: 20000<-30000)	0.96 (0.84, 1.10)	0.99 (0.76, 1.28)	0.96 (0.82, 1.11)
Annual ED volume, >30000 (ref: 20000<-30000)	1.06 (0.91, 1.24)	1.25 (0.94, 1.66)	1.01 (0.86, 1.19)
Discharge diagnosis, chronic conditions (ref: Non-MSK)	1.11 (0.99, 1.25)	1.24 (0.97, 1.60)	1.08 (0.95, 1.23)
Discharge diagnosis, injury and MSK (ref: Non-MSK)	0.91 (0.73, 1.13)	0.80 (0.58, 1.10)	0.97 (0.75, 1.26)
Discharge diagnosis, infection (ref: Non-MSK)	1.24 (1.09, 1.43)	1.08 (0.78, 1.49)	1.29 (1.12, 1.50)
Discharge diagnosis, other (ref: Non-MSK)	1.20 (1.08, 1.34)	0.91 (0.72, 1.16)	1.31 (1.15, 1.48)

Abbreviations: ED = Emergency Department; MSK = Musculoskeletal; n = number; HR = hazard ratio; VAMC = Veterans Affairs Medical Center.

© 2025 Seidenfeld J, et al. *JAMA Network Open*.

eReferences

1. Kinosian B, Wieland D, Gu X, Stallard E, Phibbs CS, Intrator O. Validation of the JEN frailty index in the National Long-Term Care Survey community population: identifying functionally impaired older adults from claims data. *BMC Health Serv Res*. Nov 29 2018;18(1):908. doi:10.1186/s12913-018-3689-2
2. Michaels AD, Meneveau MO, Hawkins RB, Charles EJ, Mehaffey JH. Socioeconomic risk-adjustment with the Area Deprivation Index predicts surgical morbidity and cost. *Surgery*. Nov 2021;170(5):1495-1500. doi:10.1016/j.surg.2021.02.016
3. Seidenfeld J, Stechuchak KM, Smith VA, et al. Frailty Explains Variation in Emergency Department Use for Older Veterans During the COVID-19 Pandemic. *Journal of geriatric emergency medicine*. 2024;5(3):10.17294/2694-4715.1080.
4. Zullig LL, Drake C, Webster A, et al. Organizational Characteristics of Hospitals Meeting STRIDE Program Adoption Benchmarks to Support Mobility for Hospitalized Persons. *INQUIRY: The Journal of Health Care Organization, Provision, and Financing*. 2024;61:00469580241274030.
5. Hastings SN, Whitson HE, Purser JL, Sloane RJ, Johnson KS. Emergency department discharge diagnosis and adverse health outcomes in older adults. *J Am Geriatr Soc*. Oct 2009;57(10):1856-61. doi:10.1111/j.1532-5415.2009.02434.x
6. HCUP Chronic Condition Indicator (CCI) for ICD-9-CM. Healthcare Cost and Utilization Project (HCUP). Agency for Healthcare Research and Quality, Rockville, MD. www.hcup-us.ahrq.gov/toolssoftware/chronic/chronic.jsp. Accessed August 26, 2024.
7. Patterson OV, Eyre H, Peterson KS, Duvall SL. From Emergency Department to Admission: mapping reasons for visit and admit diagnosis using Natural Language Processing. 2021:
8. Savova GK, Masanz JJ, Ogren PV, et al. Mayo clinical Text Analysis and Knowledge Extraction System (cTAKES): architecture, component evaluation and applications. *J Am Med Inform Assoc*. Sep-Oct 2010;17(5):507-13. doi:10.1136/jamia.2009.001560
9. Seidenfeld J, Dalton A, Vashi AA. Emergency department utilization and presenting chief complaints by Veterans living with dementia. *Acad Emerg Med*. Apr 2023;30(4):331-339. doi:10.1111/acem.14686
10. Bodenreider O. The Unified Medical Language System (UMLS): integrating biomedical terminology. *Nucleic Acids Res*. Jan 1 2004;32(Database issue):D267-70. doi:10.1093/nar/gkh061
11. Intrator O, Hiris J, Berg K, Miller SC, Mor V. The residential history file: studying nursing home residents' long-term care histories(*). *Health Serv Res*. Feb 2011;46(1 Pt 1):120-37. doi:10.1111/j.1475-6773.2010.01194.x

© 2025 Seidenfeld J, et al. *JAMA Network Open*.
