# Supplementary material for: Home Time Following Emergency Department Visits Among People With Dementia
Source: JAMA Netw Open. 2025 Dec 29;8(12):e2549154. doi: 10.1001/jamanetworkopen.2025.49154 (PMC12750257; doi:10.1001/jamanetworkopen.2025.49154)
Supplement: Supplement 2. — Data Sharing Statement [file jamanetwopen-e2549154-s002.pdf]

Data Sharing Statement

Seidenfeld. Home Time Following Emergency Department Visits Among People With Dementia. *JAMA Netw Open*. Published December 29, 2025.
doi:10.1001/jamanetworkopen.2025.49154

Data
Data available: Yes
Data types: Deidentified participant data
How to access data: De-identified data will be made available according to terms of Data Sharing Plan approved by sponsor.
When available: With publication

Supporting Documents
Document types: None

Additional Information
Who can access the data: De-identified data will be made available according to terms of Data Sharing Plan approved by sponsor.
Types of analyses: De-identified data will be made available according to terms of Data Sharing Plan approved by sponsor.
Mechanisms of data availability: De-identified data will be made available according to terms of Data Sharing Plan approved by sponsor.
